# NLRP2 Regulates Proinflammatory and Antiapoptotic Responses in Proximal Tubular Epithelial Cells

**DOI:** 10.3389/fcell.2019.00252

**Published:** 2019-10-24

**Authors:** Marianna N. Rossi, Antonia Pascarella, Valerio Licursi, Ivan Caiello, Anna Taranta, Laura R. Rega, Elena Levtchenko, Francesco Emma, Fabrizio De Benedetti, Giusi Prencipe

**Affiliations:** ^1^Department of Laboratories, Immuno-Rheumatology Research Area, Bambino Gesù Children’s Hospital, Rome, Italy; ^2^Institute for Systems Analysis and Computer Science “Antonio Ruberti,” National Research Council, Rome, Italy; ^3^Department of Biology and Biotechnologies “Charles Darwin,” Sapienza University of Rome, Rome, Italy; ^4^Laboratory of Nephrology, Department of Rare Diseases, Bambino Gesù Children’s Hospital, Rome, Italy; ^5^Department of Paediatric Nephrology & Development and Regeneration, University Hospitals Leuven, Leuven, Belgium

**Keywords:** NLRP2, proximal tubular epithelial cells, inflammation, cytokines, apoptosis, cystinosis

## Abstract

Nod-like Receptor Pyrin domain containing proteins (NLRPs) expressed by resident renal cells may contribute to the pathogenesis of multiple renal diseases. Cystinosis is a genetic disorder that affects kidney and particularly proximal tubular epithelial cells (PTEC). Here, we investigated the expression of NLRP family members in human control and cystinotic conditionally immortalized PTEC. Among all the NLRPs tested, we found that NLRP2 is highly expressed in cystinostic PTEC, but not in PTEC from healthy subjects. The NLRP2 overexpression was confirmed in primary PTEC and in kidney biopsies from cystinotic patients. In order to elucidate the role of NLRP2 in PTEC, we stably transfected control PTEC with an NLRP2-containing plasmid. We showed that NLRP2 markedly increases the production of several NF-κB regulated cytokines and chemokines. Accordingly, we demonstrated that NLRP2 interacts with IKKa and positively regulates the DNA-binding activity of p50 and p65 NF-κB, by modulating the p65 NF-κB phosphorylation status in Serine 536. Transcriptome analysis revealed that NLRP2 also upregulates the expression of profibrotic mediators and reduces that of several interferon-inducible genes. Finally, NLRP2 overexpression decreased the apoptotic cell rate. Consistently, silencing of NLRP2 by small-interfering RNA in cystinotic PTEC resulted in a significant decrease in cytokine and chemokine production as well as in an increase in the apoptosis rate. Altogether, our data reveals a previously unrecognized role for NLRP2 in regulating proinflammatory, profibrotic and antiapoptotic responses in PTEC, through NF-κB activation. Moreover, our findings unveil a novel potential mechanism involving NLRP2 overexpression in the pathogenesis of cystinosis.

## Introduction

Nod-like receptors (NLRs) are intracellular pattern-recognition receptors that sense pathogen-associated molecular patterns and endogenous damage-associated molecular patterns and, similarly to Toll-like receptors (TLRs), play a crucial role in the prompt induction of immune responses during infection as well as during tissue injury (Komada and Muruve, 2019). In humans, the NLR family consists of twenty-two proteins that are divided based on their N-terminal signaling domain, with the NLR pyrin domain-containing protein (NLRP) family comprising the largest group. The ligands and functions of most NLR family members remain to date unknown. Nevertheless, the activity of the best-understood NLRs can be classified into three categories, with some NLRs displaying more than one function: (a) regulation of IL-1β and IL-18 production by formation of the multimeric protein complex inflammasome (Malik and Kanneganti, 2017); (b) negative or positive regulation of inflammatory pathways by modulation of NF-κB activity (Thaiss et al., 2016), and (c) regulation of antiviral immunity (Moore et al., 2008; Cui et al., 2010; Lupfer and Kanneganti, 2013).

Proximal tubular epithelial cells (PTEC) are the most abundant cell type in the kidney (Liu et al., 2018). PTEC play an important role in renal repair and/or progression to chronic kidney diseases, since they not only insure tubular reabsorption in the proximal segments of the nephron, but also have endocrine and immunological functions (Schlondorff, 2008). More specifically, PTEC display innate immune characteristics, including the expression of NLRs and TLRs, that allow them to respond to a wide range of immunologic, metabolic, toxic and ischemic insults (Leemans et al., 2014; Liu et al., 2018). Indeed, upon exposure to urinary proteins as well as to TLRs/NLRs ligands and/or metabolic insults, PTEC synthesize extracellular matrix materials, growth factors and a repertoire of inflammatory molecules, including cytokines, chemokines and complement components (David et al., 1997; Gerritsma et al., 1998). The ensuing release of these bioactive mediators promotes the recruitment of immune cells, initiates tissue repair and activates adaptive immune responses aimed at regaining tissue integrity. Nevertheless, if the inflammatory reaction is inappropriate or chronic, maladaptive inflammatory and fibrogenic responses can develop, causing irreversible and progressive kidney damage (Meng et al., 2014). Indeed, a large body of evidence indicates that TLRs and NLRs expressed by different resident renal cell types, including tubular epithelial cells, may have important roles in the pathogenesis of multiple renal disorders, such as chronic kidney diseases, diabetic nephropathies, sepsis or toxic-mediated acute kidney injury (AKI) and crystal-related nephropathies (Gluba et al., 2010; Darisipudi and Knauf, 2016). Consistently, the absence of specific inflammasome components in murine models of acute or chronic kidney diseases has been demonstrated to protect mice from kidney failure (Shigeoka et al., 2010; Vilaysane et al., 2010; Zhuang et al., 2015).

Cystinosis is a rare autosomal recessive disorder caused by mutations in the *CTNS* gene, codifying for the lysosomal cystine-proton co-transporter cystinosin. PTEC are among the first affected cells in cystinosis: accumulation of cystine-crystals, changes in lysosomal morphology, oxidative stress, high susceptibility to apoptosis and dysregulation of autophagy have been demonstrated in these cells (Park et al., 2002; Laube et al., 2006; Festa et al., 2018; Luciani et al., 2018).

Based on our previous results demonstrating that cystine-crystals can act as an endogenous activator of the inflammasome and on additional data showing that endogenous uric acid crystals modulate the expression of NLRP in PTEC (Xiao et al., 2015), in this study we have investigated the expression of the best-known NLRP3 and of other members of the NLRP family in cystinotic PTEC. Having demonstrated that NLRP2 is markedly expressed in human cystinotic PTEC but not in control PTEC, we decided to investigate the functional role of NLRP2 in these cells. To this purpose, we stably transfected control PTEC with an NLRP2-containing plasmid. We found that NLRP2 upregulates the expression of proinflammatory, chemotactic and profibrotic mediators as well as reduces the apoptotic rate in PTEC, by modulating the activity of the transcription factor NF-κB. Our data reveals a previously unrecognized role for NLRP2 in PTEC and provide evidence of a novel mechanism involving NLRP2 overexpression in the pathogenesis of cystinosis.

## Materials and Methods

### Cell Culture

Two control and three cystinotic conditionally immortalized PTEC lines (ciPTEC) were kindly provided by Dr. Elena. Levtchenko and cultured as described in Wilmer et al. (2005). Cystinotic cell lines 1 and 2 carried the 57 kb deletion of the *CTNS* gene, while the cystinotic cell line 3 carried the c.518-519delCA deletion (p.Tyr173X) in exon 8 and the c.1015G>A missense mutation (p.Gly339Arg) in exon 12. Primary tubular epithelial cells were isolated from urine of five cystinotic patients and one healthy subject as described previously (Wilmer et al., 2005). The patient chronic kidney disease stage was defined, at time of urine collection, according to the CKD Work Group KDIGO 2012 clinical practice guideline (Andrassy, 2013).

To generate PTEC stably expressing NLRP2, control ciPTEC were transfected with pEZ-M68 containing full length NLRP2 EX-Z7760-M68 or pEZ-M68 empty vector (Genecopoeia) by using Lipofectamine 2000 (Life Technologies), according to the manufacturer’s procedure. After 48 hours of transfection, cells were subjected to 1 μM puromycin selection. Media was changed every 2–3 days. Cells were frozen as a polyclonal line.

For cell stimulation assays, cells were starved for 3 h in medium without serum and then stimulated with 10 ng/ml of Tumor necrosis factor alpha (TNF-α, R&D Systems), 10 mg/ml of Bovine serum albumin (BSA, Sigma), 10 μg/ml of Lipopolysaccharides (LPS, Sigma) or 10% of fetal bovine serum (FBS, GIBCO).

For treatment with NF-κB inhibitor, BAY 11-7082 (Cayman Chemicals) was added at the final concentration of 50 μM to the cell cultures. An aliquot of conditioned media was collected after 5 and 20 h since BAY 11-7082 addition for cytokine measurements. For studies of RNA expression, cells were cultured for 20 h in the presence of BAY 11-7082 and then were stimulated for 1 h with fresh medium containing TNF-α.

For apoptosis induction experiments, cells were treated with 30 ng/ml of TNF-α plus 2.5 μg/ml of Actinomycin D for 24 h. To analyze the apoptosis rate, the conditioned media of untreated or treated cells were collected and centrifuged to retrieve the floating cells. Adherent cells were detached by trypsin and combined with floating cells. After centrifugation and wash with PBS, cells were incubated with Calbiochem Annexin V-FITC apoptosis detection kit, according to the manufacturer’s procedure. Cells were analyzed by flow cytometry (FACSCanto II, BD Biosciences).

### RNA Isolation and Quantitative Real-Time PCR

Total RNA was extracted using Trizol Reagent (Ambion), and cDNAs were obtained using the Superscript Vilo kit (Invitrogen). Real-time PCR assays were performed using TaqMan Universal PCR Master Mix and gene expression assays from Applied Byosystems. Gene expression was normalized using human *HPRT1* as endogenous control. Data were analyzed with the 2^Δct^ method and are expressed as arbitrary units (AU) or as fold difference.

### Immunohistochemistry

After moist heat–induced antigen retrieval with EnVision Flex Target Retrieval Solutions High pH (DakoCytomation), 2.5-μm kidney sections were incubated with antibody to NLRP2 (sc-166584 Santa Cruz Biotechnologies) overnight at 4°C. After washing, sections were incubated with appropriate horseradish peroxidase–conjugated secondary antibodies. Chromogen detection was carried out with the DAB chromogen kit (DakoCytomation). Nuclei were counterstained with hematoxylin, followed by dehydration and mounting.

### Enzyme-Linked Immunosorbent Assays (ELISAs)

Cell culture supernatants were collected at the indicated time points and analyzed for cytokine and chemokine concentrations by using commercial ELISA kits, according to the manufacture’s procedure. IL-6 (DY206), IL-8 (DY-208), MCP-1 (DY279), CXCL1 (DGR00B), and CXCL5 (DX000) kits were all purchased from R&D Systems; Serum Amyloid A (KA0518) and G-CSF (ELH-GCSF) were purchased from Abnova and RayBiotech, respectively. The detection limit of the assays were 9.38 (IL-6), 31.3 (IL-8), 15.6 (MCP-1), 31.3 (CXCL1), 31.3 (CXCL5), 2.5 (SAA), 0.69 (G-CSF) pg/ml.

### Western Blotting and Immunoprecipitation

Cells were lysed in RIPA buffer (Cell Signaling) and protein concentration was measured with BCA Protein assay (Pierce). A total of 30 μg protein extracts were resolved by 10% SDS PAGE, transferred to nitrocellulose membranes (Amersham Life Sciences) and probed with antibody to NLRP2 (NALP2, 137569) from Abcam, NF-κB (8242), GAPDH (5174s), pS536- NF-κB (3033S), IKKα (11930S), all from Cell Signaling Technology. Blots were developed with the ECL system (Amersham Biosciences) according to the manufacturer’s protocol.

For immunoprecipitation experiments, PTEC/NLRP2 and PTEC/EV were lysed with RIPA buffer and protein concentration was measured with BCA Protein assay (Pierce). 1.5 mg of total proteins for each sample were precleared with 20 μl Dynabeads Protein A for 1 h at 4°C in rotation. Precleared lysates were incubated overnight with 1 μg of anti-NLRP2 antibody or Rabbit IgG (Invitrogen). Then 20 μl Dynabeads Protein A were added and incubated for additional 2 h. Beads were washed three times with PBS plus 0.1% Tween and once with PBS. Immunoprecipitated proteins were eluted from beads with Laemmli sample buffer (Bio-Rad 161-0747) and loaded on polyacrylamide gel to be subjected to western blot analysis.

### NF-κB DNA-Binding ELISAs

p52, p50, p65, RelB, c-Rel binding to DNA consensus sequences were assayed in nuclear lysates using the TransAm NF-κB family kit (Active Motif). Briefly, cells were lysed with NE-PER kit (Pierce) to obtain nuclear pellets. Nuclei were lysed in the Complete lysis Buffer and protein concentration was measured with Bradford assay (Bio-Rad). 50 μg of protein extracts were loaded on each well. After incubation with primary and secondary antibodies, absorbance was measured at 450 nm.

### Small Interference RNA

Proximal tubular epithelial cells were transfected using a mixture of four chemically synthesized small interference RNA (siRNA) duplex against NLRP2 (SMART pool Dharmacon cod SO-2616566G) or on target plus control pool (Dharmacon D-001810-10-05), at a final concentration of 200 nM. 72 h after transfection, cells were collected for western blot analysis or RNA extraction. For cytokine release measurements, 72 h after transfection, media were replaced with fresh media and collected 24 h later.

### RNA-Seq Trascriptomic Analysis

Total RNA was extracted and quantified as described above. RNA-seq libraries preparation and sequencing were performed by Lexogen Service using the QuantSeq 3’ mRNA-Seq Library Prep Kit FWD for Illumina by using total RNA, according to manufacturer instructions. The final libraries for single-read sequencing of 76 base pairs were carried out on an Illumina HiSeq2000. Each sample produced about 50 millions of reads.

Reads quality was evaluated using FastQC (version 0.11.2, Babraham Institute Cambridge, United Kingdom) tool. Reads were then mapped to the human Ensembl GRCh38 build reference genome using STAR version 2.5.0a (Dobin et al., 2013). The Ensembl annotation release 91 was used to build a transcriptome index that was then provided to STAR during the alignment. The average percentage of uniquely mapped reads to the human genome was 85.7%. Read distribution coverage was uniform among samples and it was evaluated with *read_distribution.py* script from RSeQC package version 2.6.4. The average percentage values of mapped reads on CDS exons, 3′UTR Exon and Introns were 24.2% ± 0.6, 48.1% ± 0.3, and 7.8% ± 0.3, respectively.

The same gene annotations were used to quantify the gene-level read counts using HTSeq-count version 0.8.0 script (Anders et al., 2015). The differential analysis for gene expression was performed using Bioconductor R version 3.4.4 package DESeq2 version 1.18.1 (Gentleman et al., 2004; Love et al., 2014).

In order to understand biological meaning of the differentially expressed genes the resulting filtered [False discovery rate (FDR) adjusted *p*-value < 0.01] genes were clustered by enrichment pathway analysis using Bioconductor R packages *clusterProfiler* (Yu et al., 2012), with annotation of Gene Ontology Database (Ashburner et al., 2000). RNA-Seq data accompanying this paper are available through Gene Expression Omnibus (GEO) repository, under accession number GSE123247.

### Statistical Analysis

Results have been reported as mean ± Standard Error Mean (SEM). Values between at least three groups were compared by the parametric ANOVA test and, if significant, pairwise comparisons were evaluated by the unpaired *t*-test. All statistical analyses were performed using GraphPad Prism V software. Statistical significance is shown as ^∗^*p* < 0.05 or ^∗∗^*p* < 0.01 or ^∗∗∗^*p* < 0.001.

## Results

### NLRP2 Is Highly Expressed in Cystinotic PTEC

**FIGURE 1:**
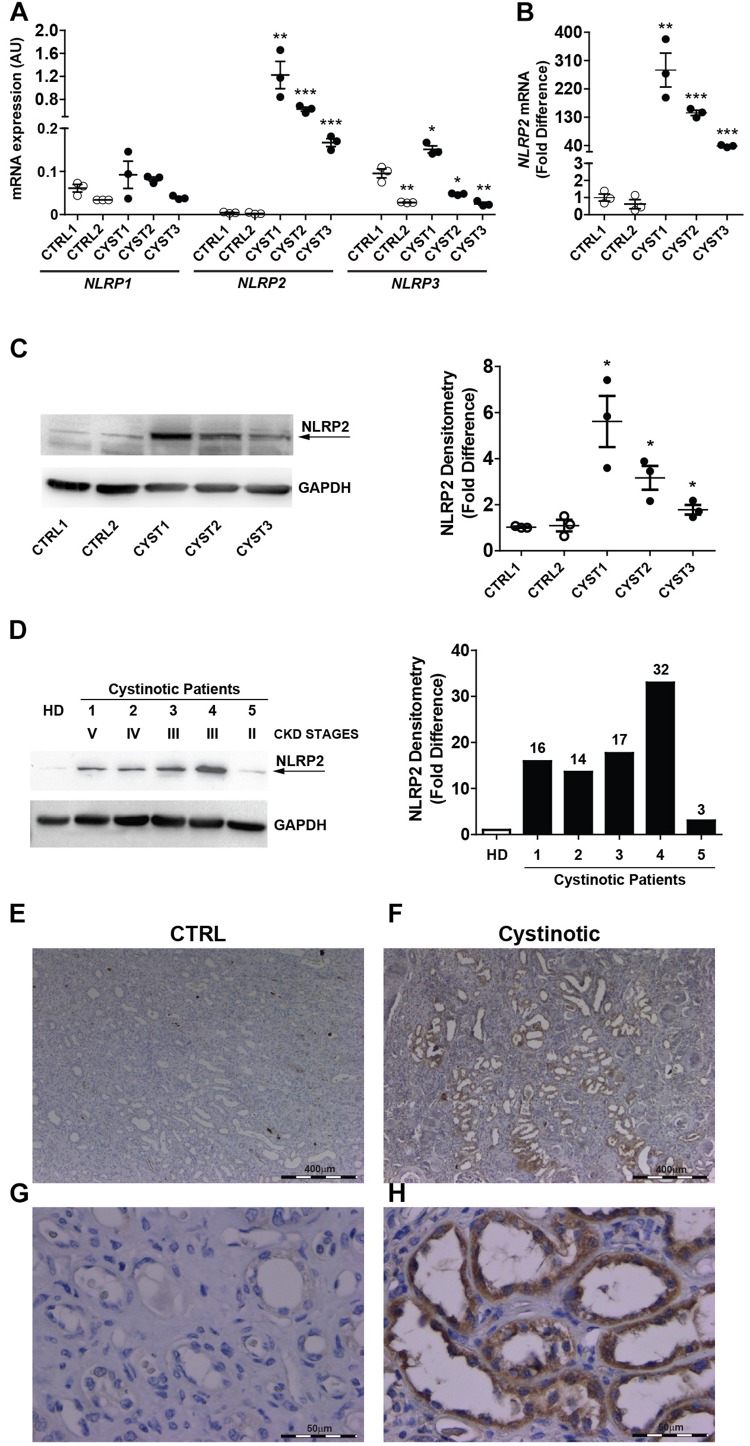
NLRP2 expression is upregulated in cystinotic proximal tubular epithelial cells. **(A,B)** *NLRP1*, *NLRP2*, and *NLRP3* mRNA expression levels were evaluated by qPCR analysis in 2 control (CTRL1 and CTRL2) and 3 cystinotic (CYST1, CYST2, and CYST3) conditionally immortalized PTEC lines. Results are obtained after normalization with the housekeeping gene *HPRT1* and are expressed as arbitrary units (AU) **(A)** and for NLRP2 as fold difference **(B)** versus the CTRL1 cell line. Differences between groups were compared using the parametric ANOVA test (*p* > 0.5 for NLRP1 and *p* < 0.0001 for NLRP2 and NLRP3) and, if significant, unpaired *t*-test was applied, comparing each cell line versus the CTRL1 cell line. **(C)** Representative western blot showing NLRP2 protein upregulation in cystinotic PTEC compared to control PTEC. The arrow indicates the specific NLRP2 upper band. GAPDH is used as loading control (left panel). Densitometric analysis of NLRP2 normalized to the corresponding band intensity of GAPDH (right graph) confirmed the upregulation of NLRP2 in cystinotic cells. The data represent mean ± SEM from at least three independent experiments and are reported as fold difference versus CTRL1 cell line. **(D)** Western Blot and densitometric analysis of NLRP2 protein levels in primary PTEC isolated from urine of 5 cystinotic patients and 1 healthy subject (HD). For each patient, the chronic kidney disease (CKD) stage at time of urine collection is reported. Immunoblot data shown are representative of more than three independent experiments. ^∗^*p* < 0.05 or ^∗∗^*p* < 0.01 or ^∗∗∗^*p* < 0.001. **(E,H)** Kidney biopsy specimens from one representative healthy subject (CTRL) and one cystinotic patient **(F,H)** were stained with an antibody against NLRP2, revealing a strong staining of NLRP2 specifically in proximal tubular epithelial cells of cystinotic kidney. **(E,F)** 5X magnification, **(G,H)** 60X magnification.

The mRNA expression of NLRP family members was analyzed by RT-qPCR in conditionally immortalized cystinotic and control PTEC. Among the 14 tested members (*NLRP1-14*), only *NLRP1*, *NLRP2*, and *NLRP3* showed detectable mRNA levels. *NLRP1* expression levels were comparable between cystinotic and control cells. *NLRP3* mRNA levels showed a mild increase only in one out of three cystinotic cell lines. Notably, we found that in all the three cystinotic PTEC analyzed the *NLRP2* gene was strongly expressed, compared to control PTEC (from 38 to 280 fold-difference), in which *NLRP2* mRNA levels were almost undetectable (Figures 1A,B). Accordingly, while the NLRP2 protein was barely expressed in control PTEC, it was detected in the cystinotic PTEC (Figure 1C). Further confirming the upregulation of NLRP2 in cystinosis, we observed markedly higher NLRP2 protein levels in primary PTEC obtained from five cystinotic patients compared to PTEC from a healthy subject (Figure 1D). Consistently, immunohistochemistry revealed strong staining for NLRP2 in PTEC of a cystinotic kidney (Figures 1F,H). In contrast, NLRP2 staining was very weak or absent in control kidney biopsies (Figures 1E,G). Collectively, these results highlights a specific and significant overexpression of NLRP2 in cystinotic PTEC *ex vivo* and *in vivo*.

### Exogenous Overexpression of NLRP2 in Control PTEC Increases the Production of Proinflammatory Cytokines and Chemokines

**FIGURE 2:**
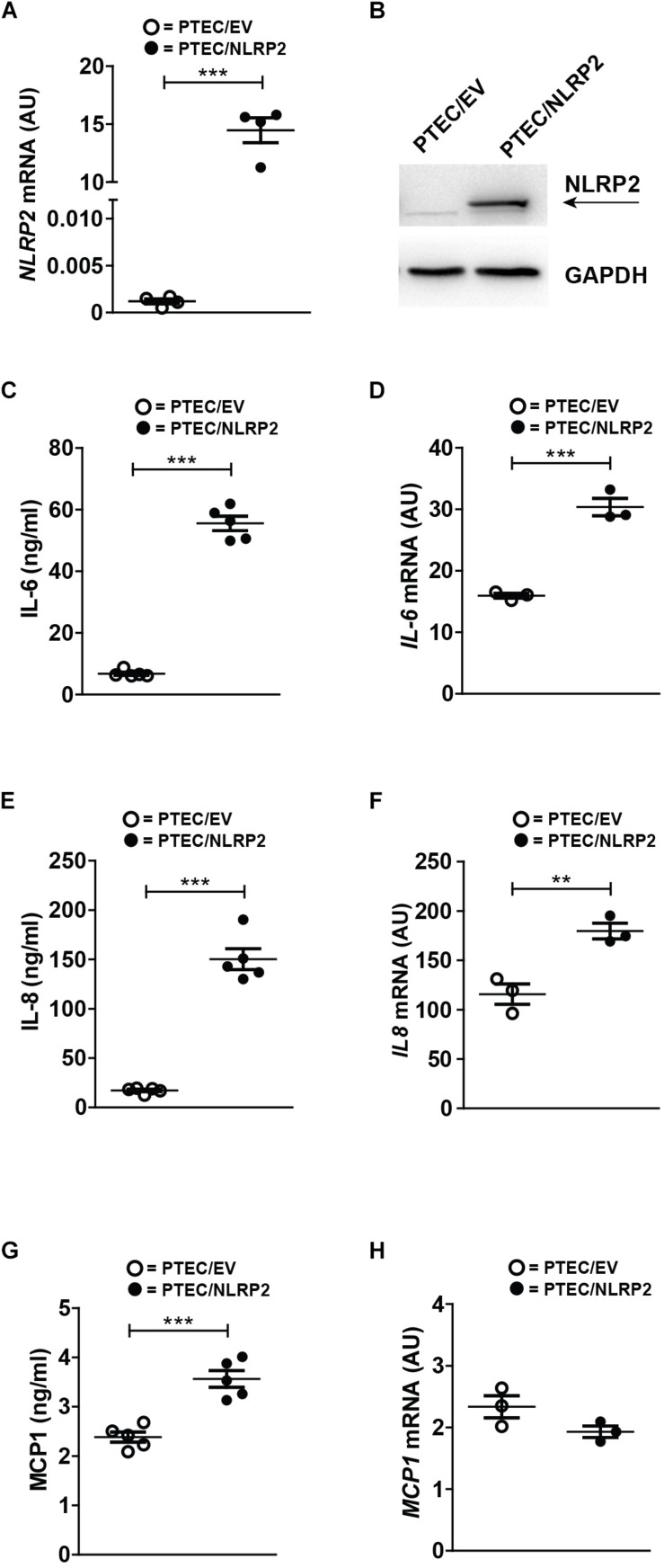
In control PTEC overexpressing NLRP2, mRNA and protein levels of proinflammatory cytokines IL-6, IL-8 and MCP-1 are increased. **(A)** *NLRP2* mRNA expression levels were evaluated by qPCR analysis in PTEC/EV and PTEC/NLRP2. Results are obtained after normalization with the housekeeping gene *HPRT1* and are expressed as arbitrary units (AU). **(B)** NLRP2 protein levels were assessed by western blot analysis in PTEC/EV and PTEC/NLRP2. GAPDH is reported as loading control. Immunoblot data shown arerepresentative of more than three independent experiments. **(C,E,G)** IL-6, IL-8, and MCP-1 levels were measured by ELISA in the conditioned media of PTEC/EV and PTEC/NLRP2 after 72 h of cell culture. **(D,F,H)** mRNA expression levels of *IL6*, *IL8*, and *MCP1* in PTEC/EV and PTEC/NLRP2 were evaluated by qPCR analysis. Results are obtained after normalization with the housekeeping gene *HPRT1* and are expressed as arbitrary units (AU). **(C–H)** The data represent mean ± SEM from at least three independent experiments. ^∗∗^*p* < 0.01 or ^∗∗∗^*p* < 0.001.

To elucidate the functional role of increased NLRP2 expression in PTEC, we established an NLRP2 overexpressing control PTEC line (PTEC/NLRP2) by transfection with a NLRP2 containing plasmid. Control PTEC transfected with an empty vector (PTEC/EV) were used as control (Figures 2A,B). Based on previously published data demonstrating that NLRP2 can form an inflammasome complex in other cell types (Bruey et al., 2004; Minkiewicz et al., 2013), we first tested if NLRP2 could form an active inflammasome in PTEC. To this end, cystinotic PTEC, PTEC/NLRP2 and PTEC/EV were stimulated for 6 or 24 h with different stimuli, including TNF-α, LPS and BSA. The inflammasome-mediated secretion of IL-1β and IL-18 was assessed after exposure of cells to the canonical NLRP3 inflammasome stimulus ATP. Levels of both IL-1β and IL-18 in culture supernatants were undetectable in all tested experimental conditions (data not shown), which excludes a role for NLRP2 in IL-1β and IL-18 mediated inflammasome release. Based on *in vitro* evidence demonstrating that NLRP2 is able to regulate inflammation by inhibiting NF-κB activity in macrophage-like THP-1 cells (Bruey et al., 2004; Fontalba et al., 2007; Tilburgs et al., 2017), we evaluated the production of NF-κB-dependent cytokines, such as IL-6, IL-8, and MCP-1 in PTEC/NLRP2. Contrary to what was expected, we found that unstimulated PTEC/NLRP2 released significantly higher levels of all analyzed cytokines, compared to PTEC/EV (Figures 2C,E,G). Consistently with these results, a significant basal increase in the mRNA levels of *IL6* and *IL8* was also observed (Figures 2D,F). No significant differences in *MCP1* mRNA levels were found (Figure 2H). These results indicate that the overexpression of NLRP2 is able to modulate the production on NF-κB -regulated cytokines in PTEC.

### NLRP2 Regulates NF-κB Activation and Interacts With IKKα

**FIGURE 3:**
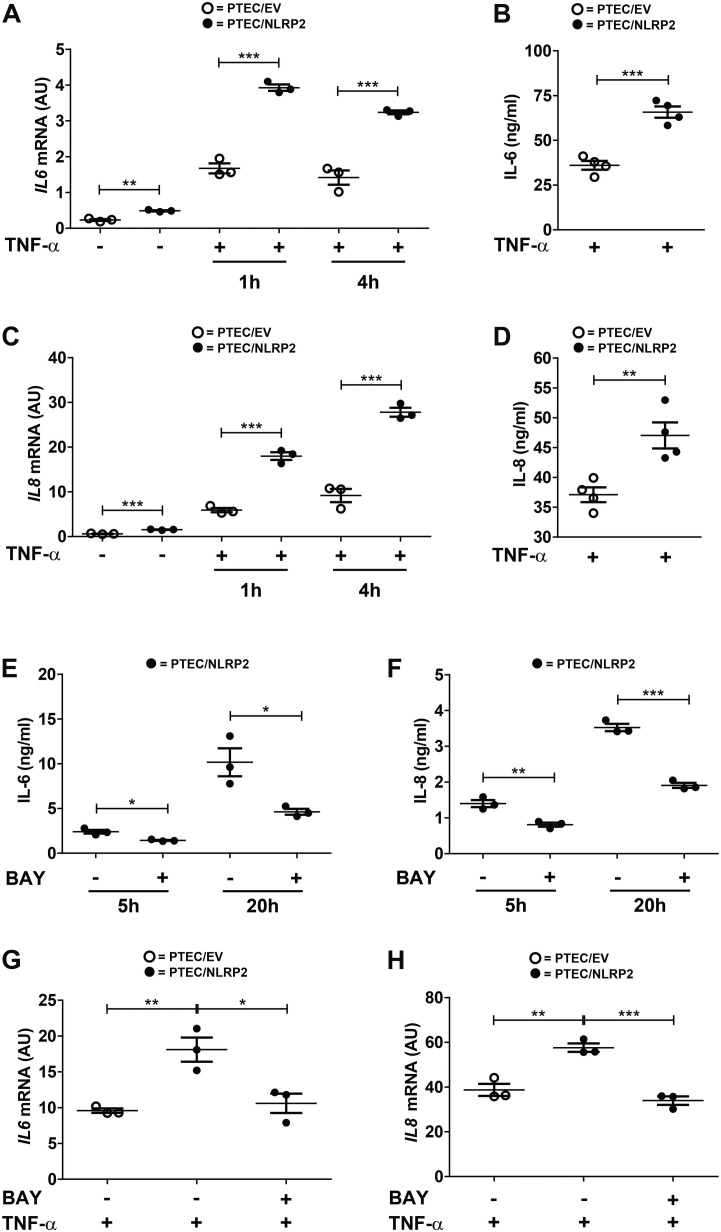
IL-6 and IL-8 levels are increased in TNF-α stimulated PTEC/NLRP2 and are decreased by NF-κB inhibition. **(A,C)** The expression levels of *IL6* and *IL8* were evaluated by qPCR analysis in PTEC/EV and PTEC/NLRP2 unstimulated and after stimulation with TNF-α (10 ng/ml) for the indicated hours (h). Results are obtained after normalization with the housekeeping gene *HPRT1* and are expressed as arbitrary units (AU). **(B,D)** PTEC/EV and PTEC/NLRP2 were stimulated with TNF-α (10 ng/ml) for 24 h and IL-6 and IL-8 were measured in the conditioned media by ELISA. **(E,F)** PTEC/NLRP2 were treated for the indicated hours (h) with 50 μM BAY 11-7082 (BAY) and conditioned media were analyzed by ELISA for IL-6 and IL-8 release. **(G,H)** PTEC/NLRP2 were untreated or treated for 20 h with 50 μM BAY 11-7082 and then stimulated with 10 ng/ml of TNF-α for 1 h. The mRNA expression levels of *IL6* and *IL8* were evaluated by qPCR analysis. Data are also compared with results obtained in TNF-α stimulated PTEC/EV. Results are obtained after normalization with the housekeeping gene *HPRT1* and are expressed as arbitrary units (AU). **(A–H)** The data represent mean ± SEM from at least three independent experiments. ^∗^*p* < 0.05, ^∗∗^*p* < 0.01, ^∗∗∗^*p* < 0.001.

**FIGURE 4:**
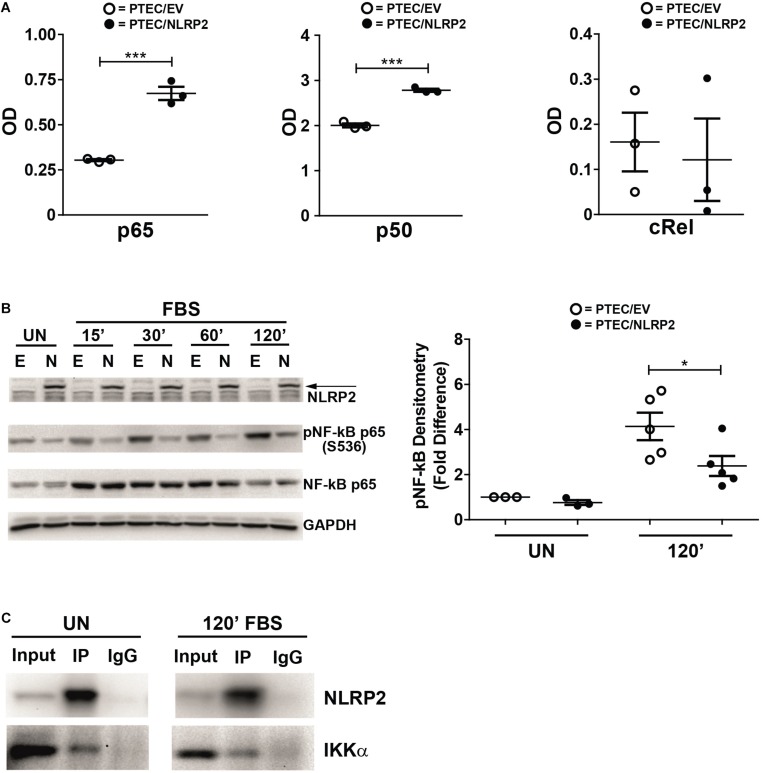
NLRP2 regulates NF-κB DNA-binding activity, reduces NF-κB p65 phosphorylation in Serine 536 and interacts with IKKα. **(A)** PTEC/EV and PTEC/NLRP2 were cultured for 3 h in media without serum. Cells were lysed and nuclear extracts were assayed with the TransAm NF-κB family kit (Active Motive) to measure the binding of p65, p50 and cRel to their DNA consensus sequences. Data are shown as mean ± SEM of at least three independent experiments. OD = optical density. **(B)** PTEC/EV (E) and PTEC/NLRP2 (N) were starved for 3 h in media without FBS and lysed (unstimulated, UN) or stimulated with 10% FBS and lysed at the indicated time points (15, 30, 60, and 120 minutes). NLRP2, phosphorylated (S536) NF-κB p65 and total NF-κB p65 protein levels were assessed by Western blot analyses. GAPDH is used as loading control. Densitometric quantification of phosphoNF-κB p65 (S536) protein levels (right graph) is reported. The data represent mean ± SEM from at least three independent experiments. **(C)** PTEC/NLRP2 were left unstimulated (UN) or were starved for 3 h and then stimulated with 10% FBS for 120 minutes (120′ FBS). Lysates were immunoprecipitated with anti-NLRP2 antibody and proteins were detected as indicated. Input = total cell lysates; IP = samples immunoprecipitated with anti-NLRP2 antibody; IgG = samples immunoprecipitated with control anti-rabbit IgG. Immunoblot data shown are representative of three independent experiments. ^∗^*p* < 0.05, ^∗∗∗^*p* < 0.001.

Our results strongly supported a role for NLRP2 in positive regulation of NF-κB transcriptional activity. In order to test this hypothesis, we evaluated whether the increased cytokine production observed in PTEC/NLRP2 was NF-κB driven. To this purpose, PTEC/NLRP2 and PTEC/EV were stimulated with TNF-α, a well-known activator of the NF-κB signaling pathway. A significant and marked increase in both mRNA (Figures 3A,C) and protein (Figures 3B,D) levels of IL-6 and IL-8 was found in PTEC/NLRP2 compared to PTEC/EV. Accordingly, when PTEC/NLRP2 were treated with a specific NF-κB inhibitor (BAY 11-7082), a strong reduction in the basal production of both IL-6 and IL-8 was observed in PTEC/NLRP2 (Figures 3E,F). In addition, we also found that treatment with the NF-κB inhibitor was able to lower *IL6* and *IL8* mRNA levels of TNF-α stimulated PTEC/NLRP2 to those observed in TNF-α stimulated PTEC/EV (Figures 3G,H), further supporting a role for NLRP2 in modulating NF-κB activation. Based on these results, we tested the DNA-binding activities of the NF-κB p65 (RelA), p50, p52, c-Rel, and RelB family members. We observed that under basal conditions the activities of p65 and p50 were significantly higher in PTEC/NLRP2 than in PTEC/EV (Figure 4A). No differences in c-Rel activity were observed (Figure 4A), while RelB and p52 activities were undetectable (data not shown). To further investigate the mechanism by which NLRP2 modulate NF-κB p65 activation, we assessed the Serine 536 (S536) phosphorylation status in serum-starved cells unstimulated or stimulated with fetal bovine serum. Lower NF-κB p65 S536 phosphorylation levels were detected in unstimulated PTEC/NLRP2 (*p* = 0.07) and in PTEC/NLRP2 stimulated for 2 h with serum (*p* = 0.03), compared to PTEC/EV (Figure 4B). Similar results were obtained also by stimulating cells with TNF-α (data not shown). Since IKKα has been identified as one of the kinases involved in p65 S536 phosphorylation (Lawrence et al., 2005), we hypothesized that NLRP2 may exert its effects in PTEC by interacting with IKKα. To verify our hypothesis, we performed co-immunoprecipitation experiments and found that NLRP2 specifically interacts with IKKα (Figure 4C). Collectively, these results demonstrated that in PTEC overexpression of NLRP2 is associated with an increased NF-κB transcriptional activity and reduced levels of S536 phosphorylated p65 NF-κB.

### NLRP2 Upregulates Biological Processes Involved in Immune Responses, Chemotaxis and Extracellular Matrix Organization and Downmodulates the Expression of Interferon-Inducible Genes

**FIGURE 5:**
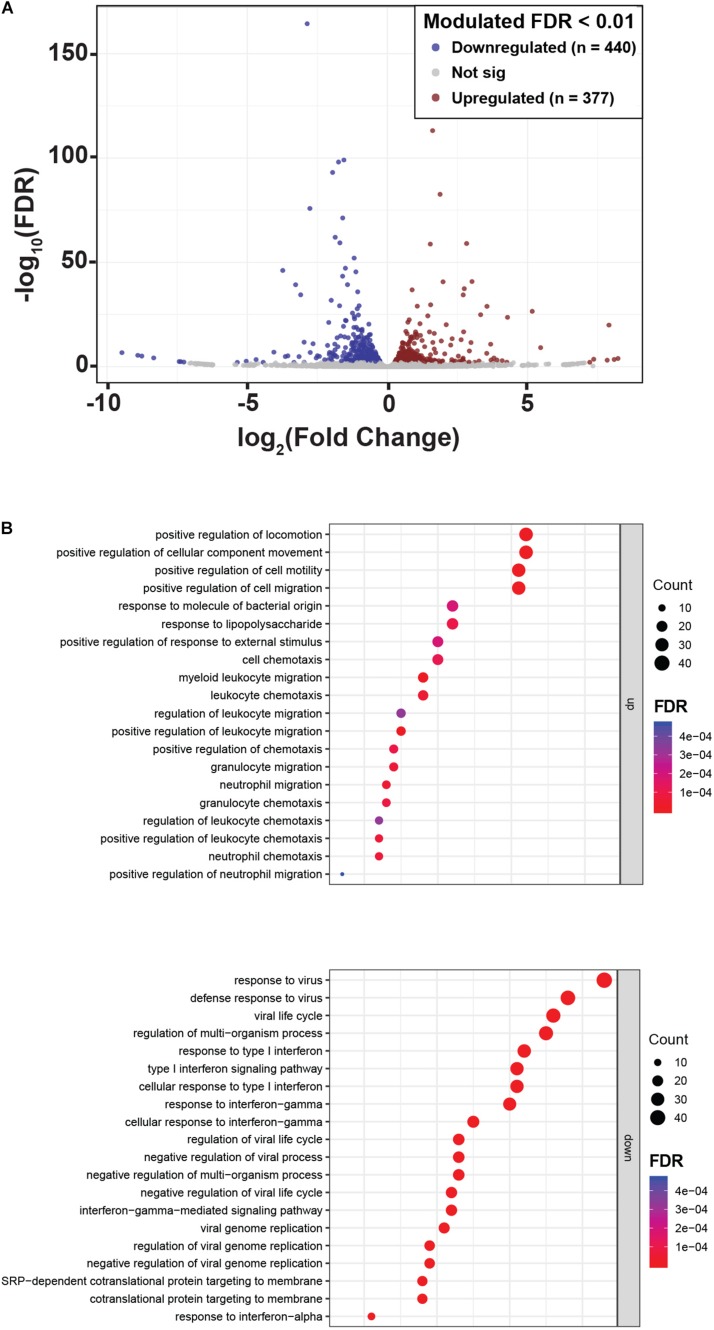
NLRP2 upregulates biological processes that are mainly involved in immune responses and chemotaxis, and downmodulates the expression of interferon inducible genes. **(A)** A volcano plot displaying the 817 differentially expressed genes in PTEC/NLRP2 versus PTEC/EV. Axes show logarithmic transformation of fold change (*x*-axis) and *p*-values (false discovery rate, FDR). Genes upregulated and downregulated in PTEC/NLRP2 with a *p*-value < 0.01 are represented in red and in blue, respectively. **(B)** Genes with differential expression were subjected to gene ontology (GO) analyses. The top 20 most significantly upregulated (upper panel) and downregulated (lower panel) biological processes are shown.

To reveal other potential genes and molecular pathways modulated by NLPR2 overexpression in PTEC, we performed an RNA-Seq transcriptomic analysis in PTEC/NLRP2 and PTEC/EV. After applying a FDR <0.01, 817 genes showed significant changes between PTEC/NLRP2 and PTEC/EV. Among them, 377 were upregulated and 440 were downregulated (Figure 5A and Supplementary Table S1). Gene Ontology biological process analyses of differentially expressed genes in PTEC/NLRP2 versus PTEC/EV were performed. The upregulated biological processes with the highest enrichment scores included items related to positive regulation of leukocyte migration and immune responses (Figure 5B). On the other hand, the downregulated gene clusters with the highest enrichment scores were all related to responses to Type I and Type II Interferons (Figure 5B). Altogether, these findings further demonstrate the role of NLRP2 in modulating the expression of a large number of mediators with a crucial role in inflammatory responses.

**FIGURE 6:**
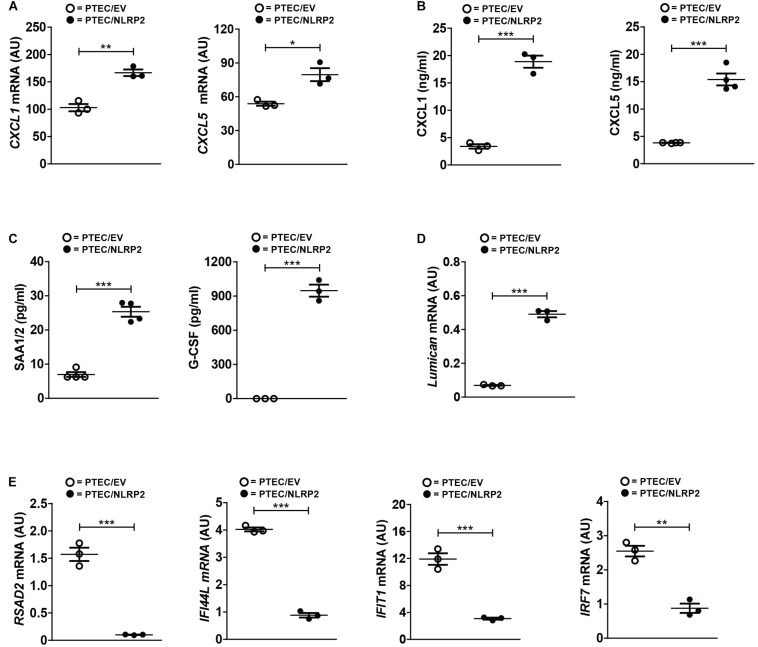
NLRP2 significantly modulates mRNA and protein levels of multiple genes involved in inflammatory and Interferon responses. **(A,D,E)** The expression levels of the indicated genes were evaluated by qPCR analysis in PTEC/EV and PTEC/NLRP2. Results are obtained after normalization with the housekeeping gene *HPRT1* and are expressed as arbitrary units (AU). Data in the graphs represent mean ± SEM of at least three independent experiments. **(B,C)** After 48 h of culture, PTEC/EV and PTEC/NLRP2 conditioned media were collected and assayed for the indicated proteins by ELISA. For G-CSF measurements, in PTEC/EV cells, levels were under the lower standard curve detection limit (0.69 pg/ml). The data represent mean ± SEM from at least three independent experiments. ^∗^*p* < 0.05 or ^∗∗^*p* < 0.01 or ^∗∗∗^*p* < 0.001.

In order to validate the differential expression of several genes from our RNA-Seq data, we first evaluated the mRNA and/or protein levels of selected upregulated genes with a known role in inflammation and chemotaxis. Six chemokines were found significantly upregulated (*CXCL1*, *CXCL2*, *CXCL3*, *CXCL5*, *CXCL6*, and *CXCL12*). Of these, we tested the mRNA and protein levels of CXCL1 and CXCL5. Not only did we confirmed their higher mRNA levels in PTEC/NLRP2 (Figure 6A), but we also observed a markedly significant increase in the release of both chemokines (Figure 6B). To further validate RNA-Seq results, in conditioned media of PTEC/NLRP2 and PTEC/EV, we measured the levels of other two factors known to be regulated by NF-κB, the major acute-phase protein serum amyloid A (*SAA1/2* genes) and the Granulocyte Colony stimulating factor (G-CSF, *CSF3* gene) (Dunn et al., 1994; Jensen and Whitehead, 1998). Consistently, we found that PTEC/NLRP2 released significantly higher amounts of both mediators (Figure 6C).

Interestingly, biological processes involved in extracellular matrix organization were also enriched in PTEC/NLRP2. Indeed, multiple genes encoding for biologically active components of the extracellular matrix that have been demonstrated to be involved in the regulation of inflammatory and fibrotic renal disorders, including type IV collagen, metalloproteases, and small leucine-rich proteoglycans (lumican, biglycan, and decorin) were upregulated in PTEC/NLRP2, compared to PTEC/EV. A significant increase in lumican gene expression in PTEC/NLRP2 was confirmed by RT-qPCR (Figure 6D).

In addition, gene ontological grouping revealed a significant downmodulation of a large number of genes induced by type I and type II Interferons (IFNs). These results have been confirmed by RT-qPCR, evaluating 4 out of the 32 IFN-inducible genes that were found to be downmodulated in the RNA-Seq analysis (Figure 6E).

Taken together, these data indicate that overexpression of NLRP2 *per se* is able to induce in control PTEC the upregulation of genes mainly related to inflammatory/chemotactic responses as well as the downregulation of IFN responses-related genes.

### NLRP2 Overexpression Exerts an Anti-apoptotic Effect in PTEC

**FIGURE 7:**
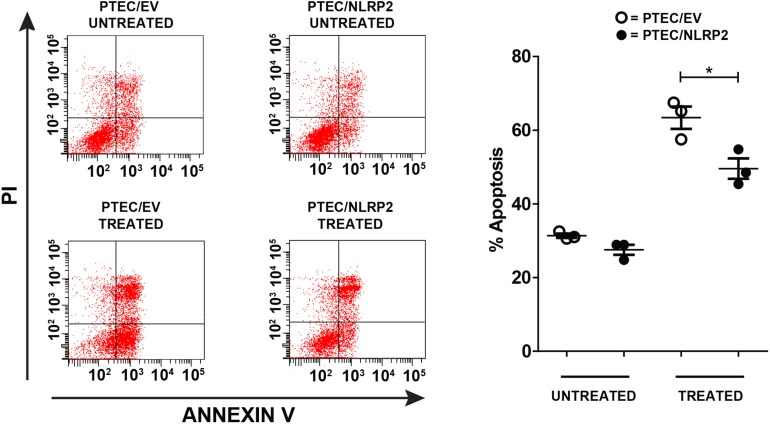
The apoptosis rate is decreased in PTEC overexpressing NLRP2. PTEC/NLRP2 and PTEC/EV were left untreated or treated with 30 ng/ml of TNF-α plus 2.5 μg/ml of Actinomycin D for 24 h. Cells were double stained with Annexin V-FITC/Propidium Iodide and apoptosis percentage was analyzed by flow cytometry. Representative scatterplots depict the Annexin V/PI stain **(left panel)**. The apoptosis rate was calculated including early and late apoptotic cells **(right panel)**. The data represent mean ± SEM from at least three independent experiments. ^∗^*p* < 0.05.

Having demonstrated that NLRP2 acts in PTEC by upregulating the NF-κB activity, we decided to evaluate whether NLRP2 overexpression influenced the apoptotic response. Indeed, the role of NF-κB in suppressing apoptosis is largely known (Luo et al., 2005). To investigate this issue, PTEC/NLRP2 and PTEC/EV were left untreated or treated with the proapoptotic stimuli Actinomycin- D and TNF-α. Consistently, PTEC/NLRP2 showed a significant lower apoptotic cell rate compared to PTEC/EV (Figure 7), suggesting an NF-κB mediated antiapoptotic role for NLRP2 in PTEC.

### *NLRP2* Silencing Reduces Cytokine and Chemokine Production as Well as Enhances Apoptosis in Cystinotic PTEC

**FIGURE 8:**
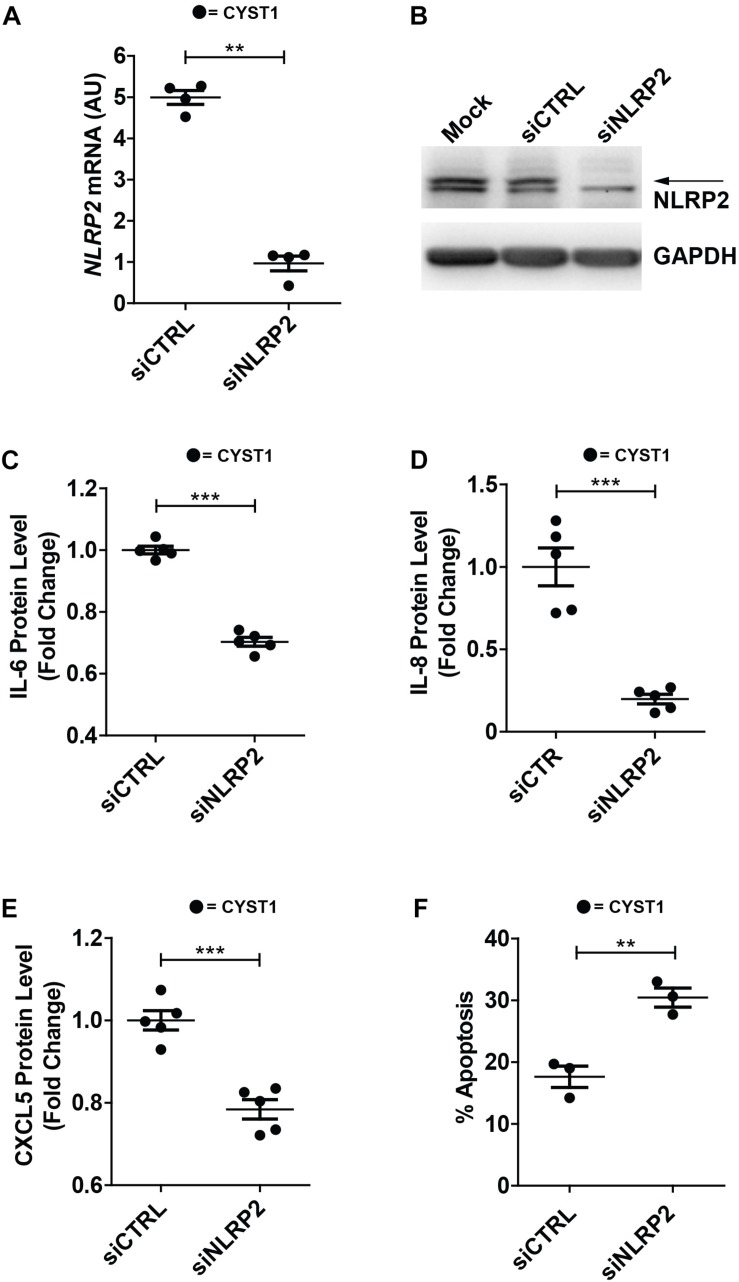
Silencing of NLRP2 in cystinotic PTEC results in decreased cytokine production and increased apoptosis rate. **(A)** *NLRP2* mRNA expression levels were assessed in cystinotic PTEC (CYST1 cell line) by qPCR analysis after 72 h of transfection with “on target plus control pool” siRNA (siCTRL) or siRNA pool against NLRP2 (siNLRP2). Results are obtained after normalization with the housekeeping gene *HPRT1* and are expressed as arbitrary units (AU). **(B)** Western blot analysis of NLRP2 protein levels in mock, siCTRL and siNLRP2 cystinotic PTEC. GAPDH is used as loading control. Immunoblot data shown are representative of more than three independent experiments. **(C–E)** IL-6, IL-8 and CXCL5 levels were analyzed by ELISA in the conditioned media of cystinotic PTEC transfected for 96 h with siCTRL and siNLRP2. **(F)** The apoptosis rate was calculated in siCTRL and siNLRP2 cystinotic PTEC treated with 30 ng/ml of TNFα plus 2.5 μg/ml of Actinomycin D for 24 h. Cells were double stained with Annexin V-FITC/Propidium Iodide and apoptosis percentage was analyzed by flow cytometry. The apoptosis rate was calculated including early and late apoptotic cells. **(A–F)** The data represent mean ± SEM from at least three independent experiments. ^∗∗^*p* < 0.05, ^∗∗∗^*p* < 0.001.

Finally, in order to verify whether high NLRP2 levels in cystinotic cells regulated the expression of inflammatory-related genes, we performed experiments of *NLRP2* small interfering RNA (siRNA) in conditionally immortalized cystinotic PTEC. Although we observed a marked decrease in NLRP2 mRNA (>five-fold decrease) and protein levels (Figures 8A,B), *NLRP2* expression remained significantly higher than that observed in control PTEC. Nevertheless, we found that cystinotic PTEC silenced for *NLRP2* released significantly lower amounts of IL-6, IL-8 and CXCL5, compared to control siRNA cells (Figures 8C–E), supporting the proinflammatory role of NLRP2 in cystinotic cells. Moreover, consistent with our previous results, we found a significant increase in the apoptosis rate in NLRP2-silenced cystinotic PTEC compared to control siRNA-silenced cells exposed to proapoptotic stimuli (Figure 8F). Altogether, these results support the pro-inflammatory and antiapoptotic role of NLRP2 in cystinotic cells.

## Discussion

In this study, we report the overexpression of NLRP2 *ex vivo* and *in vivo* in cystinotic PTEC and identify a previously unrecognized role for NLRP2 in the regulation of proinflammatory, chemotactic, fibrotic and apoptotic responses in these cells.

NLR proteins are cytosolic receptors with key roles in innate immune responses and tissue homeostasis (Kufer and Sansonetti, 2011). Few data on NLRP2 expression and functions are available. Studies on NLRP2 knock out mice revealed that NLRP2 is selectively expressed in ovaries and, while it is dispensable for innate and adaptive immunity, it plays a key role in early embryogenesis and fertility (Kuchmiy et al., 2016; Mahadevan et al., 2017). Accordingly, a recent meta-analysis of endometrial gene expression profiles of patients with embryo implantation failure demonstrated that *NLPR2* mRNA levels are significantly reduced in these patients and that inflammatory responses are significantly downmodulated, providing evidence for a proinflammatory role of NLRP2 *in vivo* (Li et al., 2017). Conversely, results obtained *in vitro* in different cell types, such as THP1 macrophages or astrocytes, in which NLRP2 has been exogenously expressed, suggest a role of NLRP2 in repressing the activity of NF-κB (Bruey et al., 2004; Fontalba et al., 2007). In the present study, we have shown that NLRP2 is highly expressed in cystinotic PTEC, as demonstrated by analyses performed in conditionally immortalized cells as well as in primary cells and kidney biopsies from cystinotic patients, while it is almost undetectable in PTEC derived from healthy subjects. Moreover, we demonstrated that the exogenous expression of NLRP2 in control PTEC causes a marked increase in the expression and production of NF-κB driven cytokines, such as IL-6, IL-8, CXCL1, CXCL5. Accordingly, we found that NLRP2 upregulates the NF-κB p65 and p50 DNA-binding activities. These apparently conflicting results on the activatory and inhibitory activities of NLRP2 could be explained by different functionalities of NLRP2 in different cell types, as already demonstrated for other NLR family members (Li et al., 2018; Luan et al., 2018). In addition, most of the studies on NLRPs have been performed on immune cells and the full spectrum of NLRP functions in different tissues and cells is still limited. In this context, our study extends the knowledge of NLRP2 functions in PTEC.

NF-κB transcriptional activity is modulated through different mechanisms, among which phosphorylation is one of the best characterized (Christian et al., 2016). S536 is one of the best-understood phosphorylation targets in the transactivation domain of p65. Although several studies demonstrated that S536 phosphorylation is required for the activation and nuclear translocation of NF-κB, an important role for S536 phosphorylation in inhibiting NF-κB signaling has been recently demonstrated (Lawrence et al., 2005; Pradere et al., 2016), suggesting that the effects of S536 phosphorylation would appear to depend mainly on the physiological context. Indeed, phosphorylation of p65 S536 has been shown to promote the proteasomal degradation of p65 and, therefore, to limit NF-κB transcriptional responses. Moreover, Lawrence et al. (2005) identified IKKα as the kinase responsible for the phosphorylation of p65 S536 in macrophages. Consistent with this evidence, we found that the phosphorylation status of p65 S536 is decreased in unstimulated and serum-stimulated PTEC/NLRP2 and is associated with an increased NF-κB activity. In addition, we also found that NLRP2 interacts with IKKα, leading us to speculate that NLRP2 could modulate the NF-κB transcriptional activity by interacting with IKKα and interfering with the phosphorylation of p65 in S536.

To better understand the physiopathological role of NLRP2 in PTEC, we performed an RNA-Seq transcriptomic analysis comparing control cells transfected with the NLRP2 gene or with an EV. We found that NLRP2 *per se* is able to modulate the expression of a large number of genes involved in biological processes mainly related to inflammatory responses, chemotaxis as well as extracellular matrix organization. These results are in accordance with the evidence that NF-κB is the master regulator of the inflammatory responses as well as regulates the transcription of genes controlling proliferation and cell death (Lawrence, 2009). Although kidney cell-type specific NF-κB functions remain unknown, an increasing body of evidence demonstrated the involvement of this transcription factor in the pathogenesis of renal diseases, such as ischemia/reperfusion AKI, sepsis-induced AKI, glomerulonephritis, or allogenic renal transplantation (Sanz et al., 2010). Interestingly, a recent study performed in a mouse model with specific tubular epithelia-NF-κB suppression showed that post-ischemic NF-κB activation in PTEC aggravates tubular injury and exacerbates a dysregulated immune response by increasing cytokine and chemokine production (Marko et al., 2016). To date, the role of inflammation in the pathogenesis of cystinosis is not clearly established. Nevertheless, kidneys of *Ctns*^–/–^ mice are characterized by tubular atrophy and interstitial inflammatory infiltrates. In addition, a preliminary observational analysis on a large number of cystinotic patients has shown an improved renal outcome in patients treated with the anti-inflammatory drug indomethacin that (Emma et al., 2016), incidentally, has been demonstrated to inhibit the NF-κB activity (Sung et al., 2004), therefore supporting a pathogenic role of inflammation in the progression of renal disease. In the light of these observations, our data demonstrating the upregulation of NLRP2 in cystinotic PTEC and the reduction of proinflammatory cytokines and chemokines in cystinotic PTEC silenced for NLRP2 shed a new light on mechanisms causing inflammation in cystinosis.

A recent study has demonstrated that NLRP2 negatively regulates antiviral immunity by inhibiting the TANK-binding kinase 1 (TBK1)-dependent IFN signaling in human embryonic kidney 293T cells (Yang et al., 2018). In agreement with these results, we observed that also in PTEC NLRP2 markedly reduced mRNA expression levels of several IFNs-regulated genes. Our study did not address specifically the molecular mechanism involved in the NLPR2 response. However, it has been shown that IKKα is also critically involved in the activation of the interferon regulatory factor (IRF) 3 and IRF7, which are the major transcription factors in the regulation of IFN-inducible genes (Hoshino et al., 2006; Wang et al., 2008). The exact role of the activation of the IFN signaling pathway in PTEC needs to be established in future studies. Nevertheless, a common side effect of high-dose IFN therapy is the renal impairment that has been demonstrated to be associated with tubular cell dysfunction and tubular cell death (Izzedine et al., 2005). Likewise, IFNs involvement has been demonstrated in ischemic reperfusion–triggered pro-apoptotic pathways in PTEC (Lechner et al., 2008).

Whether upregulation of NLRP2 is restricted to cystinotic PTEC or is a common response in tubular damage needs further studies. In this respect, it is interesting to notice that a whole genome gene expression study performed in a large number of deceased donor kidney biopsies prior to engraftment has shown increased NLRP2 expression levels associated with acute tubular damage, further supporting a role of NLRP2 in proximal tubular dysfunction (Perco et al., 2009). These findings led us to speculate a model in which damaged/injured PTEC upregulate NLRP2 expression that, in turn, increases the production of cytokines, chemokines and extracellular matrix components and protects cells from apoptosis. Hypothetically, this response is aimed at recruiting immune cells, activating innate and adaptive immune responses, and promoting cell and tissue repair. In chronic or genetic diseases, including cystinosis, the sustained overexpression of NLRP2 could contribute to the progression of renal failure by creating a vicious inflammatory cycle.

Taken together, our findings demonstrated a novel role for NLRP2 in regulating proinflammatory, profibrotic and antiapoptotic responses in PTEC. Furthermore, our data shed light on a potential mechanism involving NLRP2 overexpression in the pathogenesis of cystinosis and other tubulopathies, providing the rationale for novel targeted anti-inflammatory therapeutic strategies that may complement presently available conventional therapies.

## Data Availability Statement

The datasets generated for this study can be found in the RNA-Seq data accompanying this manuscript are available through Gene Expression Omnibus (GEO) repository, under accession number GSE123247.

## Ethics Statement

The studies involving human participants were reviewed and approved by the Bambino Gesù Children’s Hospital Institutional Ethical Committees. Written informed consent to participate in this study was provided by the participants’ legal guardian/next of kin.

## Author Contributions

MR, FD, and GP designed the study. MR, AP, and IC carried out the experiments. VL analyzed the RNA-seq data. AT, LR, and EL provided essential reagents and techniques for this study. MR, AP, and GP analyzed the data. FE and EL contributed to the study design. FD and GP supervised the research design. GP supervised the laboratory activities and drafted the manuscript. All authors contributed to the manuscript writing and approved the final version of the manuscript.

## Conflict of Interest

The authors declare that the research was conducted in the absence of any commercial or financial relationships that could be construed as a potential conflict of interest.
