# Supplementary material for: NLRP2 Regulates Proinflammatory and Antiapoptotic Responses in Proximal Tubular Epithelial Cells
Source: Front Cell Dev Biol. 2019 Oct 24;7:252. doi: 10.3389/fcell.2019.00252 (PMC6822264; doi:10.3389/fcell.2019.00252)
Supplement: Supplementary file 1 [file Table_1.DOCX]

**Supplementary table 1. List of modulated genes in PTEC/NLRP2.** A list of all the genes upregulated and downregulated in PTEC/NLRP2 versus PTEC/EV (FDR< 0.01) is reported.

|  | | | | | | |
| --- | --- | --- | --- | --- | --- | --- |
| **Ensembl ID** | **Symbol** | **Entrez ID** | **Mean of normalized counts of all samples** | **log2 Fold Change** | **pvalue** | **FDR** |
| **ENSG00000214548** | MEG3 | 55384 | 65.9236526 | -9.47 | 2.06E-09 | 1.85E-07 |
| **ENSG00000172179** | PRL | 5617 | 44.473952 | -8.90 | 5.14E-08 | 3.53E-06 |
| **ENSG00000100234** | TIMP3 | 7078 | 40.3207271 | -8.76 | 1.26E-07 | 7.96E-06 |
| **ENSG00000114948** | ADAM23 | 8745 | 30.0362079 | -8.34 | 1.26E-06 | 6.27E-05 |
| **ENSG00000108813** | DLX4 | 1748 | 16.0035643 | -7.43 | 1.36E-04 | 3.42E-03 |
| **ENSG00000203930** | LINC00632 | 286411 | 15.4029597 | -7.37 | 1.57E-04 | 3.86E-03 |
| **ENSG00000236432** | LOC654841 | 654841 | 14.2563322 | -7.26 | 2.64E-04 | 5.96E-03 |
| **ENSG00000136542** | GALNT5 | 11227 | 20.7970903 | -5.35 | 4.11E-04 | 8.58E-03 |
| **ENSG00000020633** | RUNX3 | 864 | 25.4396173 | -5.04 | 8.56E-05 | 2.30E-03 |
| **ENSG00000135750** | KCNK1 | 3775 | 39.5325518 | -4.68 | 1.03E-05 | 3.91E-04 |
| **ENSG00000171873** | ADRA1D | 146 | 22.7937046 | -4.43 | 3.70E-04 | 7.87E-03 |
| **ENSG00000175928** | LRRN1 | 57633 | 85.3992744 | -4.05 | 1.14E-09 | 1.07E-07 |
| **ENSG00000134321** | RSAD2 | 91543 | 577.151274 | -3.73 | 7.12E-50 | 7.76E-47 |
| **ENSG00000271503** | CCL5 | 6352 | 76.2339299 | -3.64 | 2.28E-07 | 1.38E-05 |
| **ENSG00000168077** | SCARA3 | 51435 | 94.5205216 | -3.55 | 9.72E-08 | 6.31E-06 |
| **ENSG00000154639** | CXADR | 1525 | 33.9539898 | -3.33 | 4.16E-04 | 8.65E-03 |
| **ENSG00000134326** | CMPK2 | 129607 | 713.3717 | -3.27 | 6.80E-43 | 5.30E-40 |
| **ENSG00000141052** | MYOCD | 93649 | 80.4551169 | -3.15 | 2.48E-07 | 1.49E-05 |
| **ENSG00000272398** | CD24 | 100133941 | 644.283401 | -3.09 | 5.56E-38 | 3.64E-35 |
| **ENSG00000171564** | FGB | 2244 | 52.6077707 | -2.97 | 3.19E-04 | 6.98E-03 |
| **ENSG00000165449** | SLC16A9 | 220963 | 205.042958 | -2.97 | 1.01E-14 | 1.72E-12 |
| **ENSG00000130303** | BST2 | 684 | 6235.02889 | -2.86 | 2.76E-169 | 4.52E-165 |
| **ENSG00000117152** | RGS4 | 5999 | 1658.03229 | -2.77 | 6.20E-80 | 1.45E-76 |
| **ENSG00000008196** | TFAP2B | 7021 | 151.909143 | -2.75 | 1.71E-09 | 1.56E-07 |
| **ENSG00000186340** | THBS2 | 7058 | 253.084835 | -2.65 | 6.58E-14 | 9.97E-12 |
| **ENSG00000159251** | ACTC1 | 70 | 102.067389 | -2.54 | 1.48E-06 | 7.16E-05 |
| **ENSG00000117069** | ST6GALNAC5 | 81849 | 156.820745 | -2.50 | 8.72E-08 | 5.73E-06 |
| **ENSG00000165025** | SYK | 6850 | 146.422882 | -2.43 | 1.93E-07 | 1.18E-05 |
| **ENSG00000048740** | CELF2 | 10659 | 146.543084 | -2.24 | 1.35E-05 | 4.90E-04 |
| **ENSG00000240694** | PNMA2 | 10687 | 434.961425 | -2.13 | 4.71E-13 | 6.31E-11 |
| **ENSG00000110446** | SLC15A3 | 51296 | 122.709599 | -2.10 | 1.14E-05 | 4.25E-04 |
| **ENSG00000113389** | NPR3 | 4883 | 719.51315 | -2.09 | 1.79E-24 | 6.35E-22 |
| **ENSG00000137673** | MMP7 | 4316 | 235.598365 | -2.07 | 1.73E-08 | 1.33E-06 |
| **ENSG00000185070** | FLRT2 | 23768 | 244.671036 | -2.07 | 1.61E-09 | 1.47E-07 |
| **ENSG00000185885** | IFITM1 | 8519 | 173.71832 | -2.01 | 1.90E-06 | 8.85E-05 |
| **ENSG00000132274** | TRIM22 | 10346 | 1967.31975 | -2.00 | 2.48E-35 | 1.50E-32 |
| **ENSG00000003147** | ICA1 | 3382 | 131.467816 | -1.98 | 6.89E-05 | 1.91E-03 |
| **ENSG00000018236** | CNTN1 | 1272 | 291.032036 | -1.98 | 2.23E-09 | 2.00E-07 |
| **ENSG00000157601** | MX1 | 4599 | 13328.1871 | -1.96 | 2.58E-97 | 8.45E-94 |
| **ENSG00000169184** | MN1 | 4330 | 233.411376 | -1.93 | 2.22E-07 | 1.35E-05 |
| **ENSG00000136869** | TLR4 | 7099 | 237.871753 | -1.92 | 1.27E-08 | 9.91E-07 |
| **ENSG00000144583** | MARCH4 | 57574 | 463.936131 | -1.88 | 1.02E-13 | 1.49E-11 |
| **ENSG00000135114** | OASL | 8638 | 435.588672 | -1.87 | 7.72E-11 | 8.47E-09 |
| **ENSG00000137959** | IFI44L | 10964 | 4454.77585 | -1.86 | 5.34E-66 | 9.70E-63 |
| **ENSG00000104332** | SFRP1 | 6422 | 306.788448 | -1.86 | 5.22E-08 | 3.57E-06 |
| **ENSG00000154175** | ABI3BP | 25890 | 263.717521 | -1.80 | 3.59E-07 | 2.06E-05 |
| **ENSG00000069482** | GAL | 51083 | 1113.06911 | -1.79 | 6.88E-17 | 1.36E-14 |
| ENSG00000276107 | |  | 826.864077 | -1.77 | 2.09E-12 | 2.61E-10 |
| **ENSG00000115461** | IGFBP5 | 3488 | 10760.6266 | -1.74 | 2.08E-102 | 8.49E-99 |
| **ENSG00000185745** | IFIT1 | 3434 | 2894.12085 | -1.71 | 1.17E-32 | 6.39E-30 |
| **ENSG00000111335** | OAS2 | 4939 | 4702.94518 | -1.70 | 2.78E-63 | 4.55E-60 |
| **ENSG00000086289** | EPDR1 | 54749 | 785.730171 | -1.67 | 8.15E-18 | 1.88E-15 |
| **ENSG00000197457** | STMN3 | 50861 | 166.927002 | -1.64 | 6.00E-05 | 1.71E-03 |
| **ENSG00000078401** | EDN1 | 1906 | 4855.76257 | -1.60 | 4.44E-47 | 4.27E-44 |
| **ENSG00000126709** | IFI6 | 2537 | 25031.1638 | -1.60 | 2.63E-75 | 5.39E-72 |
| **ENSG00000138650** | PCDH10 | 57575 | 294.936785 | -1.59 | 1.45E-07 | 9.05E-06 |
| **ENSG00000170961** | HAS2 | 3037 | 1581.13394 | -1.57 | 1.44E-22 | 4.63E-20 |
| **ENSG00000118523** | CTGF | 1490 | 23782.6024 | -1.55 | 1.63E-103 | 8.89E-100 |
| **ENSG00000184979** | USP18 | 11274 | 703.600435 | -1.55 | 1.58E-09 | 1.45E-07 |
| **ENSG00000074527** | NTN4 | 59277 | 221.452418 | -1.55 | 8.44E-05 | 2.28E-03 |
| **ENSG00000137965** | IFI44 | 10561 | 1130.94851 | -1.53 | 2.21E-17 | 4.95E-15 |
| **ENSG00000119917** | IFIT3 | 3437 | 5956.406 | -1.50 | 5.44E-51 | 6.36E-48 |
| **ENSG00000089127** | OAS1 | 4938 | 2221.68155 | -1.50 | 1.64E-25 | 6.24E-23 |
| **ENSG00000119922** | IFIT2 | 3433 | 841.138573 | -1.50 | 5.45E-12 | 6.65E-10 |
| **ENSG00000198729** | PPP1R14C | 81706 | 269.333395 | -1.49 | 5.75E-06 | 2.32E-04 |
| **ENSG00000162409** | PRKAA2 | 5563 | 481.340513 | -1.47 | 6.01E-07 | 3.30E-05 |
| **ENSG00000107201** | DDX58 | 23586 | 1810.21419 | -1.47 | 2.28E-25 | 8.48E-23 |
| **ENSG00000198780** | FAM169A | 26049 | 300.040179 | -1.46 | 1.86E-06 | 8.71E-05 |
| **ENSG00000187608** | ISG15 | 9636 | 35334.9694 | -1.42 | 5.94E-43 | 4.86E-40 |
| **ENSG00000169851** | PCDH7 | 5099 | 312.721428 | -1.42 | 2.72E-06 | 1.21E-04 |
| **ENSG00000178776** | C5orf46 | 389336 | 453.592904 | -1.42 | 2.18E-08 | 1.63E-06 |
| **ENSG00000204642** | HLA-F | 3134 | 1090.57217 | -1.40 | 6.54E-17 | 1.30E-14 |
| **ENSG00000038295** | TLL1 | 7092 | 272.721867 | -1.39 | 4.12E-05 | 1.23E-03 |
| **ENSG00000099284** | H2AFY2 | 55506 | 244.027182 | -1.39 | 1.96E-04 | 4.65E-03 |
| **ENSG00000168016** | TRANK1 | 9881 | 311.302331 | -1.38 | 3.25E-05 | 1.01E-03 |
| **ENSG00000078018** | MAP2 | 4133 | 288.524164 | -1.37 | 2.03E-05 | 6.85E-04 |
| **ENSG00000057294** | PKP2 | 5318 | 364.545523 | -1.36 | 7.69E-05 | 2.10E-03 |
| **ENSG00000140937** | CDH11 | 1009 | 667.37308 | -1.35 | 2.28E-08 | 1.70E-06 |
| **ENSG00000115267** | IFIH1 | 64135 | 665.525147 | -1.34 | 6.40E-09 | 5.28E-07 |
| **ENSG00000129514** | FOXA1 | 3169 | 249.079402 | -1.32 | 1.28E-04 | 3.26E-03 |
| **ENSG00000139117** | CPNE8 | 144402 | 229.138693 | -1.31 | 4.32E-04 | 8.95E-03 |
| **ENSG00000213626** | LBH | 81606 | 517.223452 | -1.30 | 3.86E-08 | 2.72E-06 |
| **ENSG00000165655** | ZNF503 | 84858 | 374.59772 | -1.30 | 1.61E-05 | 5.67E-04 |
| **ENSG00000076356** | PLXNA2 | 5362 | 726.423979 | -1.28 | 7.25E-11 | 8.01E-09 |
| **ENSG00000180914** | OXTR | 5021 | 1607.58242 | -1.27 | 2.05E-13 | 2.91E-11 |
| **ENSG00000212724** | KRTAP2-3 | 730755 | 307.416632 | -1.27 | 4.89E-04 | 9.81E-03 |
| **ENSG00000177409** | SAMD9L | 219285 | 1141.61972 | -1.26 | 1.65E-14 | 2.64E-12 |
| **ENSG00000106785** | TRIM14 | 9830 | 3222.05493 | -1.25 | 4.45E-29 | 2.08E-26 |
| **ENSG00000128833** | MYO5C | 55930 | 331.145177 | -1.24 | 1.35E-05 | 4.90E-04 |
| **ENSG00000183486** | MX2 | 4600 | 1227.30567 | -1.23 | 2.83E-14 | 4.36E-12 |
| **ENSG00000176697** | BDNF | 627 | 1731.58046 | -1.22 | 1.04E-11 | 1.24E-09 |
| **ENSG00000122592** | HOXA7 | 3204 | 1102.63822 | -1.22 | 5.69E-13 | 7.56E-11 |
| **ENSG00000205413** | SAMD9 | 54809 | 2521.37333 | -1.21 | 1.27E-18 | 3.15E-16 |
| **ENSG00000138675** | FGF5 | 2250 | 864.632071 | -1.20 | 3.23E-10 | 3.29E-08 |
| **ENSG00000111186** | WNT5B | 81029 | 4379.6976 | -1.20 | 4.84E-22 | 1.49E-19 |
| **ENSG00000107281** | NPDC1 | 56654 | 1095.03614 | -1.19 | 5.20E-12 | 6.39E-10 |
| **ENSG00000142089** | IFITM3 | 10410 | 38581.3375 | -1.19 | 7.10E-56 | 8.93E-53 |
| **ENSG00000160932** | LY6E | 4061 | 5019.69277 | -1.18 | 2.54E-27 | 1.07E-24 |
| **ENSG00000095739** | BAMBI | 25805 | 373.139874 | -1.18 | 2.85E-05 | 9.06E-04 |
| **ENSG00000148677** | ANKRD1 | 27063 | 5052.30539 | -1.17 | 2.78E-26 | 1.11E-23 |
| **ENSG00000201998** | SNORA23 | 677808 | 291.92093 | -1.16 | 2.29E-04 | 5.30E-03 |
| **ENSG00000132109** | TRIM21 | 6737 | 583.797551 | -1.16 | 1.52E-07 | 9.45E-06 |
| **ENSG00000162599** | NFIA | 4774 | 487.522365 | -1.15 | 1.01E-06 | 5.20E-05 |
| **ENSG00000152778** | IFIT5 | 24138 | 1804.74422 | -1.15 | 1.17E-14 | 1.97E-12 |
| **ENSG00000100342** | APOL1 | 8542 | 1997.78484 | -1.14 | 4.34E-17 | 9.25E-15 |
| **ENSG00000185880** | TRIM69 | 140691 | 1102.55513 | -1.13 | 2.27E-10 | 2.36E-08 |
| **ENSG00000068079** | IFI35 | 3430 | 1765.87211 | -1.13 | 3.09E-15 | 5.38E-13 |
| **ENSG00000146674** | IGFBP3 | 3486 | 15195.9238 | -1.12 | 3.43E-49 | 3.51E-46 |
| **ENSG00000135919** | SERPINE2 | 5270 | 4478.79407 | -1.11 | 4.21E-22 | 1.32E-19 |
| **ENSG00000065809** | FAM107B | 83641 | 815.285744 | -1.10 | 2.53E-08 | 1.87E-06 |
| **ENSG00000204175** | GPRIN2 | 9721 | 399.261934 | -1.10 | 2.67E-05 | 8.59E-04 |
| **ENSG00000172183** | ISG20 | 3669 | 1689.07581 | -1.09 | 5.06E-10 | 5.02E-08 |
| **ENSG00000120875** | DUSP4 | 1846 | 444.447181 | -1.08 | 2.20E-05 | 7.28E-04 |
| **ENSG00000117525** | F3 | 2152 | 6000.35128 | -1.08 | 3.48E-31 | 1.73E-28 |
| **ENSG00000162654** | GBP4 | 115361 | 337.374625 | -1.07 | 1.62E-04 | 3.96E-03 |
| **ENSG00000130813** | C19orf66 | 55337 | 1999.21761 | -1.06 | 6.25E-13 | 8.24E-11 |
| **ENSG00000135480** | KRT7 | 3855 | 13198.2745 | -1.06 | 1.92E-39 | 1.31E-36 |
| **ENSG00000167772** | ANGPTL4 | 51129 | 961.923172 | -1.05 | 6.38E-08 | 4.31E-06 |
| **ENSG00000138496** | PARP9 | 83666 | 3128.84739 | -1.04 | 5.48E-21 | 1.63E-18 |
| **ENSG00000151414** | NEK7 | 140609 | 1408.26922 | -1.04 | 8.94E-08 | 5.85E-06 |
| **ENSG00000130589** | HELZ2 | 85441 | 3503.58324 | -1.04 | 2.94E-17 | 6.49E-15 |
| **ENSG00000188313** | PLSCR1 | 5359 | 5999.16101 | -1.04 | 4.00E-28 | 1.77E-25 |
| **ENSG00000138642** | HERC6 | 55008 | 2779.30658 | -1.03 | 4.41E-17 | 9.25E-15 |
| **ENSG00000137198** | GMPR | 2766 | 1707.3386 | -1.03 | 1.02E-10 | 1.11E-08 |
| **ENSG00000148798** | INA | 9118 | 482.860644 | -1.03 | 5.99E-05 | 1.71E-03 |
| **ENSG00000111859** | NEDD9 | 4739 | 1255.06587 | -1.02 | 4.48E-11 | 5.02E-09 |
| **ENSG00000130433** | CACNG6 | 59285 | 329.386057 | -1.02 | 3.86E-04 | 8.12E-03 |
| **ENSG00000111331** | OAS3 | 4940 | 8500.56814 | -1.01 | 1.10E-32 | 6.21E-30 |
| **ENSG00000162407** | PLPP3 | 8613 | 605.647441 | -1.01 | 3.54E-06 | 1.54E-04 |
| **ENSG00000149573** | MPZL2 | 10205 | 455.776308 | -1.00 | 3.66E-05 | 1.12E-03 |
| **ENSG00000050344** | NFE2L3 | 9603 | 1194.20527 | -1.00 | 2.96E-07 | 1.74E-05 |
| **ENSG00000127528** | KLF2 | 10365 | 774.109338 | -1.00 | 9.74E-07 | 5.04E-05 |
| **ENSG00000156587** | UBE2L6 | 9246 | 1986.77188 | -0.99 | 1.48E-14 | 2.40E-12 |
| **ENSG00000111110** | PPM1H | 57460 | 359.846281 | -0.99 | 2.78E-04 | 6.23E-03 |
| **ENSG00000136111** | TBC1D4 | 9882 | 857.901673 | -0.98 | 7.88E-08 | 5.24E-06 |
| **ENSG00000154642** | C21orf91 | 54149 | 743.125211 | -0.97 | 1.35E-05 | 4.90E-04 |
| **ENSG00000176165** | FOXG1 | 2290 | 718.713426 | -0.97 | 9.08E-07 | 4.73E-05 |
| **ENSG00000135046** | ANXA1 | 301 | 1901.4492 | -0.96 | 7.36E-10 | 7.12E-08 |
| **ENSG00000101384** | JAG1 | 182 | 1848.28599 | -0.96 | 4.39E-13 | 5.93E-11 |
| **ENSG00000169252** | ADRB2 | 154 | 572.055149 | -0.96 | 1.30E-04 | 3.29E-03 |
| **ENSG00000176692** | FOXC2 | 2303 | 1969.59528 | -0.95 | 2.22E-11 | 2.55E-09 |
| **ENSG00000178685** | PARP10 | 84875 | 604.23474 | -0.95 | 2.84E-05 | 9.02E-04 |
| **ENSG00000150687** | PRSS23 | 11098 | 3935.74924 | -0.94 | 4.48E-18 | 1.05E-15 |
| **ENSG00000184497** | TMEM255B | 348013 | 762.535506 | -0.94 | 1.73E-06 | 8.20E-05 |
| **ENSG00000185201** | IFITM2 | 10581 | 6150.08844 | -0.94 | 9.47E-21 | 2.76E-18 |
| **ENSG00000196116** | TDRD7 | 23424 | 2007.40881 | -0.93 | 4.27E-13 | 5.83E-11 |
| **ENSG00000156113** | KCNMA1 | 3778 | 2141.30254 | -0.93 | 1.30E-13 | 1.86E-11 |
| **ENSG00000206190** | ATP10A | 57194 | 1044.8733 | -0.93 | 2.62E-08 | 1.92E-06 |
| **ENSG00000160886** | LY6K | 54742 | 640.888981 | -0.93 | 1.59E-05 | 5.61E-04 |
| **ENSG00000125398** | SOX9 | 6662 | 974.009117 | -0.93 | 4.58E-06 | 1.91E-04 |
| **ENSG00000131018** | SYNE1 | 23345 | 381.880473 | -0.93 | 4.52E-04 | 9.26E-03 |
| **ENSG00000133106** | EPSTI1 | 94240 | 1254.01485 | -0.92 | 5.70E-09 | 4.73E-07 |
| **ENSG00000168394** | TAP1 | 6890 | 3269.38405 | -0.92 | 7.33E-10 | 7.12E-08 |
| **ENSG00000164161** | HHIP | 64399 | 4706.34169 | -0.91 | 4.13E-20 | 1.19E-17 |
| **ENSG00000134363** | FST | 10468 | 1298.57124 | -0.90 | 3.93E-08 | 2.76E-06 |
| **ENSG00000204262** | COL5A2 | 1290 | 5052.1837 | -0.90 | 4.21E-21 | 1.27E-18 |
| **ENSG00000118515** | SGK1 | 6446 | 943.511243 | -0.90 | 7.44E-07 | 3.96E-05 |
| **ENSG00000181381** | DDX60L | 91351 | 1049.82918 | -0.89 | 1.56E-07 | 9.62E-06 |
| **ENSG00000167644** | C19orf33 | 64073 | 3784.60973 | -0.89 | 4.04E-16 | 7.60E-14 |
| **ENSG00000136997** | MYC | 4609 | 1169.64385 | -0.88 | 3.42E-06 | 1.50E-04 |
| **ENSG00000184254** | ALDH1A3 | 220 | 440.585892 | -0.88 | 3.71E-04 | 7.89E-03 |
| **ENSG00000136999** | NOV | 4856 | 615.997348 | -0.87 | 1.12E-04 | 2.91E-03 |
| **ENSG00000141574** | SECTM1 | 6398 | 704.485198 | -0.87 | 6.86E-05 | 1.91E-03 |
| **ENSG00000179862** | CITED4 | 163732 | 892.791323 | -0.87 | 6.17E-06 | 2.47E-04 |
| **ENSG00000106692** | FKTN | 2218 | 619.947443 | -0.87 | 7.31E-05 | 2.00E-03 |
| **ENSG00000152078** | TMEM56 | 148534 | 1070.65846 | -0.87 | 4.01E-07 | 2.29E-05 |
| **ENSG00000136848** | DAB2IP | 153090 | 939.6 | -0.87 | 1.15E-06 | 5.82E-05 |
| **ENSG00000162616** | DNAJB4 | 11080 | 1739.35781 | -0.86 | 1.96E-06 | 9.06E-05 |
| **ENSG00000164442** | CITED2 | 10370 | 3587.78844 | -0.86 | 3.35E-13 | 4.64E-11 |
| **ENSG00000107984** | 1 DKK | 22943 | 3707.52074 | -0.85 | 1.66E-15 | 2.99E-13 |
| **ENSG00000138646** | HERC5 | 51191 | 2706.2312 | -0.85 | 2.72E-12 | 3.38E-10 |
| **ENSG00000181751** | C5orf30 | 90355 | 1208.94047 | -0.85 | 4.92E-07 | 2.73E-05 |
| **ENSG00000140280** | LYSMD2 | 256586 | 726.344715 | -0.85 | 3.65E-05 | 1.12E-03 |
| **ENSG00000081923** | ATP8B1 | 5205 | 1921.63671 | -0.84 | 1.55E-10 | 1.64E-08 |
| **ENSG00000135899** | SP110 | 3431 | 2456.69187 | -0.84 | 6.15E-11 | 6.84E-09 |
| **ENSG00000152661** | GJA1 | 2697 | 4247.93496 | -0.84 | 1.35E-14 | 2.23E-12 |
| **ENSG00000129353** | SLC44A2 | 57153 | 2807.85737 | -0.83 | 3.79E-13 | 5.21E-11 |
| **ENSG00000185507** | IRF7 | 3665 | 3254.36767 | -0.82 | 9.25E-13 | 1.20E-10 |
| **ENSG00000099860** | GADD45B | 4616 | 4342.46944 | -0.82 | 7.84E-17 | 1.53E-14 |
| **ENSG00000249784** | SCARNA22 | 677770 | 3232.47372 | -0.82 | 6.89E-07 | 3.72E-05 |
| **ENSG00000112414** | ADGRG6 | 57211 | 1063.62101 | -0.81 | 4.13E-06 | 1.76E-04 |
| **ENSG00000145349** | CAMK2D | 817 | 1037.82911 | -0.80 | 4.30E-06 | 1.82E-04 |
| **ENSG00000126778** | SIX1 | 6495 | 777.504663 | -0.80 | 4.05E-05 | 1.22E-03 |
| ENSG00000181097 | |  | 595.092338 | -0.80 | 2.19E-04 | 5.08E-03 |
| **ENSG00000137628** | DDX60 | 55601 | 918.672926 | -0.80 | 1.00E-05 | 3.82E-04 |
| **ENSG00000142627** | EPHA2 | 1969 | 5899.33554 | -0.80 | 6.68E-09 | 5.49E-07 |
| **ENSG00000187024** | PTRH1 | 138428 | 876.264423 | -0.77 | 4.86E-05 | 1.42E-03 |
| **ENSG00000179242** | CDH4 | 1002 | 736.85735 | -0.77 | 1.15E-04 | 2.96E-03 |
| **ENSG00000178078** | STAP2 | 55620 | 877.246497 | -0.77 | 2.19E-05 | 7.25E-04 |
| **ENSG00000198121** | LPAR1 | 1902 | 809.888684 | -0.77 | 8.42E-05 | 2.28E-03 |
| **ENSG00000140853** | NLRC5 | 84166 | 762.197289 | -0.76 | 8.53E-05 | 2.29E-03 |
| **ENSG00000183691** | NOG | 9241 | 966.990567 | -0.76 | 2.45E-05 | 7.92E-04 |
| **ENSG00000151729** | SLC25A4 | 291 | 670.377327 | -0.76 | 2.12E-04 | 4.94E-03 |
| **ENSG00000090372** | STRN4 | 29888 | 1002.68314 | -0.76 | 9.65E-05 | 2.56E-03 |
| **ENSG00000135069** | PSAT1 | 29968 | 3768.13406 | -0.76 | 1.18E-11 | 1.39E-09 |
| **ENSG00000141664** | ZCCHC2 | 54877 | 822.177412 | -0.76 | 7.11E-05 | 1.96E-03 |
| **ENSG00000197961** | ZNF121 | 7675 | 1136.37798 | -0.76 | 1.09E-05 | 4.09E-04 |
| **ENSG00000113070** | HBEGF | 1839 | 2218.82347 | -0.76 | 1.22E-08 | 9.68E-07 |
| **ENSG00000158122** | AAED1 | 195827 | 742.3698 | -0.76 | 3.02E-04 | 6.65E-03 |
| **ENSG00000115339** | GALNT3 | 2591 | 1199.09236 | -0.75 | 2.96E-06 | 1.30E-04 |
| **ENSG00000131773** | KHDRBS3 | 10656 | 843.971079 | -0.74 | 1.79E-04 | 4.29E-03 |
| **ENSG00000137203** | TFAP2A | 7020 | 940.949618 | -0.74 | 3.59E-05 | 1.10E-03 |
| **ENSG00000196739** | COL27A1 | 85301 | 962.609666 | -0.74 | 3.84E-05 | 1.16E-03 |
| **ENSG00000173821** | RNF213 | 57674 | 7991.00598 | -0.74 | 4.36E-17 | 9.25E-15 |
| **ENSG00000166582** | CENPV | 201161 | 3175.02303 | -0.73 | 4.29E-07 | 2.42E-05 |
| **ENSG00000101347** | SAMHD1 | 25939 | 2766.97638 | -0.73 | 5.06E-10 | 5.02E-08 |
| **ENSG00000169439** | SDC2 | 6383 | 1909.87484 | -0.72 | 5.94E-08 | 4.03E-06 |
| **ENSG00000136783** | NIPSNAP3A | 25934 | 1146.81722 | -0.72 | 2.22E-05 | 7.31E-04 |
| **ENSG00000204525** | HLA-C | 3107 | 8401.33454 | -0.72 | 7.27E-15 | 1.25E-12 |
| **ENSG00000214944** | ARHGEF28 | 64283 | 1396.24721 | -0.72 | 2.82E-06 | 1.25E-04 |
| **ENSG00000168575** | SLC20A2 | 6575 | 1745.76482 | -0.71 | 1.07E-05 | 4.02E-04 |
| **ENSG00000122643** | NT5C3A | 51251 | 1661.13677 | -0.71 | 2.07E-06 | 9.53E-05 |
| **ENSG00000073712** | FERMT2 | 10979 | 8066.9611 | -0.71 | 5.62E-17 | 1.16E-14 |
| **ENSG00000188290** | HES4 | 57801 | 1867.28565 | -0.70 | 2.18E-05 | 7.25E-04 |
| **ENSG00000163840** | DTX3L | 151636 | 7042.84286 | -0.70 | 1.54E-12 | 1.96E-10 |
| **ENSG00000112787** | FBRSL1 | 57666 | 840.987444 | -0.69 | 1.74E-04 | 4.18E-03 |
| **ENSG00000105287** | PRKD2 | 25865 | 1307.73268 | -0.69 | 4.38E-05 | 1.30E-03 |
| **ENSG00000168273** | SMIM4 | 440957 | 3114.47674 | -0.69 | 2.93E-10 | 2.99E-08 |
| **ENSG00000161091** | MFSD12 | 126321 | 4545.09633 | -0.69 | 1.65E-07 | 1.01E-05 |
| **ENSG00000135045** | C9orf40 | 55071 | 1569.65293 | -0.69 | 3.81E-06 | 1.64E-04 |
| **ENSG00000117228** | GBP1 | 2633 | 4001.6197 | -0.69 | 7.46E-07 | 3.96E-05 |
| **ENSG00000160439** | RDH13 | 112724 | 784.68476 | -0.69 | 3.26E-04 | 7.12E-03 |
| **ENSG00000173193** | PARP14 | 54625 | 5707.78376 | -0.68 | 1.12E-10 | 1.21E-08 |
| **ENSG00000172159** | FRMD3 | 257019 | 902.867356 | -0.68 | 4.24E-04 | 8.81E-03 |
| **ENSG00000105559** | PLEKHA4 | 57664 | 1385.87432 | -0.68 | 4.95E-06 | 2.03E-04 |
| **ENSG00000114395** | CYB561D2 | 11068 | 965.426591 | -0.67 | 1.49E-04 | 3.69E-03 |
| **ENSG00000159403** | C1R | 715 | 909.098559 | -0.67 | 1.68E-04 | 4.07E-03 |
| **ENSG00000164086** | DUSP7 | 1849 | 1665.79484 | -0.67 | 7.23E-05 | 1.99E-03 |
| **ENSG00000147027** | TMEM47 | 83604 | 905.083825 | -0.67 | 2.07E-04 | 4.85E-03 |
| **ENSG00000070950** | RAD18 | 56852 | 1099.52869 | -0.67 | 8.06E-05 | 2.19E-03 |
| **ENSG00000153071** | DAB2 | 1601 | 6228.80393 | -0.67 | 3.70E-11 | 4.20E-09 |
| **ENSG00000148110** | MFSD14B | 84641 | 1452.24083 | -0.67 | 1.21E-05 | 4.46E-04 |
| **ENSG00000171451** | DSEL | 92126 | 1852.9554 | -0.66 | 4.40E-07 | 2.47E-05 |
| **ENSG00000166710** | B2M | 567 | 93582.3962 | -0.66 | 1.23E-23 | 4.20E-21 |
| **ENSG00000154447** | SH3RF1 | 57630 | 1812.65211 | -0.66 | 8.02E-06 | 3.10E-04 |
| **ENSG00000240065** | PSMB9 | 5698 | 4202.43778 | -0.65 | 9.83E-10 | 9.35E-08 |
| **ENSG00000234745** | HLA-B | 3106 | 56927.6401 | -0.65 | 2.24E-18 | 5.39E-16 |
| **ENSG00000050405** | LIMA1 | 51474 | 9337.03696 | -0.64 | 8.43E-14 | 1.25E-11 |
| **ENSG00000164045** | CDC25A | 993 | 2066.18477 | -0.63 | 1.06E-06 | 5.41E-05 |
| **ENSG00000123975** | CKS2 | 1164 | 3335.17447 | -0.63 | 4.27E-05 | 1.27E-03 |
| **ENSG00000174136** | RGMB | 285704 | 1622.3562 | -0.63 | 1.01E-04 | 2.64E-03 |
| **ENSG00000150630** | VEGFC | 7424 | 1144.50932 | -0.62 | 3.70E-04 | 7.87E-03 |
| **ENSG00000105137** | SYDE1 | 85360 | 2354.85856 | -0.62 | 2.71E-07 | 1.61E-05 |
| **ENSG00000110092** | CCND1 | 595 | 27379.9908 | -0.62 | 1.09E-15 | 2.00E-13 |
| **ENSG00000187957** | DNER | 92737 | 3578.54915 | -0.62 | 3.47E-08 | 2.51E-06 |
| **ENSG00000130402** | ACTN4 | 81 | 53968.7108 | -0.61 | 1.48E-18 | 3.61E-16 |
| **ENSG00000172936** | MYD88 | 4615 | 987.049909 | -0.61 | 3.48E-04 | 7.47E-03 |
| **ENSG00000135040** | NAA35 | 60560 | 1316.74696 | -0.61 | 3.13E-04 | 6.87E-03 |
| **ENSG00000130382** | MLLT1 | 4298 | 1246.53533 | -0.60 | 1.30E-04 | 3.29E-03 |
| **ENSG00000142871** | CYR61 | 3491 | 73667.7973 | -0.60 | 1.44E-10 | 1.52E-08 |
| **ENSG00000168994** | PXDC1 | 221749 | 2392.26015 | -0.60 | 2.74E-06 | 1.22E-04 |
| **ENSG00000002549** | LAP3 | 51056 | 2292.72555 | -0.60 | 1.75E-05 | 6.09E-04 |
| **ENSG00000179021** | C3orf38 | 285237 | 1096.64353 | -0.59 | 2.60E-04 | 5.90E-03 |
| **ENSG00000117318** | ID3 | 3399 | 8307.88434 | -0.59 | 1.18E-10 | 1.26E-08 |
| **ENSG00000067066** | SP100 | 6672 | 3201.12537 | -0.59 | 3.26E-06 | 1.43E-04 |
| **ENSG00000116717** | GADD45A | 1647 | 3953.47417 | -0.59 | 1.14E-07 | 7.28E-06 |
| **ENSG00000275832** | ARHGAP23 | 57636 | 3253.49309 | -0.59 | 6.49E-08 | 4.35E-06 |
| **ENSG00000074590** | NUAK1 | 9891 | 2369.85269 | -0.59 | 3.51E-06 | 1.53E-04 |
| **ENSG00000177051** | FBXO46 | 23403 | 1309.56132 | -0.59 | 1.40E-04 | 3.53E-03 |
| **ENSG00000006015** | REX1BD | 55049 | 2953.6396 | -0.58 | 2.32E-06 | 1.05E-04 |
| **ENSG00000138772** | ANXA3 | 306 | 3380.65401 | -0.58 | 5.37E-08 | 3.66E-06 |
| **ENSG00000175793** | SFN | 2810 | 3206.18188 | -0.58 | 1.16E-07 | 7.41E-06 |
| **ENSG00000163513** | TGFBR2 | 7048 | 2965.4879 | -0.58 | 6.94E-06 | 2.74E-04 |
| **ENSG00000104522** | TSTA3 | 7264 | 1879.81663 | -0.58 | 2.70E-05 | 8.67E-04 |
| **ENSG00000169908** | TM4SF1 | 4071 | 2035.49082 | -0.58 | 1.26E-05 | 4.62E-04 |
| **ENSG00000233016** | SNHG7 | 84973 | 1727.8609 | -0.58 | 4.32E-05 | 1.28E-03 |
| **ENSG00000175592** | FOSL1 | 8061 | 1854.54241 | -0.58 | 1.44E-05 | 5.16E-04 |
| **ENSG00000180340** | FZD2 | 2535 | 1718.37125 | -0.57 | 2.10E-04 | 4.91E-03 |
| **ENSG00000104884** | ERCC2 | 2068 | 2169.94266 | -0.57 | 4.19E-06 | 1.78E-04 |
| **ENSG00000134986** | NREP | 9315 | 1286.88855 | -0.57 | 2.86E-04 | 6.37E-03 |
| **ENSG00000117143** | UAP1 | 6675 | 3099.08833 | -0.57 | 2.44E-07 | 1.47E-05 |
| **ENSG00000141873** | SLC39A3 | 29985 | 1453.14787 | -0.57 | 1.31E-04 | 3.32E-03 |
| **ENSG00000148120** | C9orf3 | 84909 | 1791.5821 | -0.57 | 2.08E-05 | 7.00E-04 |
| **ENSG00000180801** | ARSJ | 79642 | 2327.51065 | -0.57 | 2.47E-06 | 1.12E-04 |
| **ENSG00000160888** | IER2 | 9592 | 6442.10796 | -0.57 | 1.79E-09 | 1.61E-07 |
| **ENSG00000142227** | EMP3 | 2014 | 2728.7404 | -0.57 | 1.74E-06 | 8.21E-05 |
| **ENSG00000117877** | CD3EAP | 10849 | 1501.06279 | -0.57 | 1.67E-04 | 4.06E-03 |
| **ENSG00000137801** | THBS1 | 7057 | 87267.9191 | -0.57 | 2.80E-13 | 3.95E-11 |
| **ENSG00000100596** | SPTLC2 | 9517 | 2901.44508 | -0.57 | 3.63E-06 | 1.57E-04 |
| **ENSG00000106829** | TLE4 | 7091 | 2315.66738 | -0.56 | 2.73E-05 | 8.71E-04 |
| **ENSG00000182871** | COL18A1 | 80781 | 2716.8169 | -0.56 | 2.32E-06 | 1.05E-04 |
| **ENSG00000136810** | TXN | 7295 | 10083.4806 | -0.56 | 7.06E-09 | 5.75E-07 |
| **ENSG00000196449** | YRDC | 79693 | 1828.81535 | -0.55 | 6.35E-05 | 1.79E-03 |
| **ENSG00000170581** | STAT2 | 6773 | 1879.34117 | -0.55 | 5.42E-05 | 1.57E-03 |
| **ENSG00000166833** | NAV2 | 89797 | 2000.1944 | -0.55 | 3.21E-05 | 1.01E-03 |
| **ENSG00000197614** | MFAP5 | 8076 | 1417.83385 | -0.55 | 2.88E-04 | 6.40E-03 |
| **ENSG00000103335** | PIEZO1 | 9780 | 3544.29665 | -0.54 | 1.89E-05 | 6.45E-04 |
| **ENSG00000107372** | ZFAND5 | 7763 | 2906.19994 | -0.54 | 1.76E-05 | 6.11E-04 |
| **ENSG00000123609** | NMI | 9111 | 1814.88931 | -0.54 | 6.41E-05 | 1.80E-03 |
| **ENSG00000162244** | RPL29 | 6159 | 6761.21216 | -0.54 | 9.69E-10 | 9.27E-08 |
| **ENSG00000108679** | LGALS3BP | 3959 | 8155.44984 | -0.54 | 1.45E-07 | 9.05E-06 |
| **ENSG00000070404** | FSTL3 | 10272 | 4087.56199 | -0.53 | 1.27E-07 | 8.00E-06 |
| **ENSG00000179820** | MYADM | 91663 | 17456.2224 | -0.53 | 1.87E-12 | 2.36E-10 |
| **ENSG00000121060** | TRIM25 | 7706 | 5428.36615 | -0.53 | 1.06E-06 | 5.41E-05 |
| **ENSG00000160710** | ADAR | 103 | 9386.08414 | -0.53 | 3.56E-08 | 2.55E-06 |
| **ENSG00000135318** | NT5E | 4907 | 13821.7665 | -0.53 | 5.98E-12 | 7.24E-10 |
| **ENSG00000120217** | CD274 | 29126 | 4082.8241 | -0.53 | 2.02E-05 | 6.83E-04 |
| **ENSG00000143702** | CEP170 | 9859 | 1452.27318 | -0.53 | 4.71E-04 | 9.53E-03 |
| **ENSG00000119681** | LTBP2 | 4053 | 6054.83569 | -0.52 | 9.56E-08 | 6.23E-06 |
| **ENSG00000129911** | KLF16 | 83855 | 2303.79563 | -0.52 | 1.84E-05 | 6.33E-04 |
| **ENSG00000152558** | TMEM123 | 114908 | 5822.80911 | -0.52 | 1.56E-07 | 9.62E-06 |
| **ENSG00000132153** | DHX30 | 22907 | 3416.67551 | -0.52 | 2.89E-06 | 1.28E-04 |
| **ENSG00000147854** | UHRF2 | 115426 | 2326.7187 | -0.51 | 2.15E-05 | 7.19E-04 |
| **ENSG00000132382** | MYBBP1A | 10514 | 2194.28536 | -0.51 | 6.40E-05 | 1.80E-03 |
| **ENSG00000108639** | SYNGR2 | 9144 | 5884.95438 | -0.51 | 1.76E-08 | 1.34E-06 |
| **ENSG00000064313** | TAF2 | 6873 | 1791.55112 | -0.51 | 1.34E-04 | 3.39E-03 |
| **ENSG00000163659** | TIPARP | 25976 | 1793.17277 | -0.51 | 4.87E-04 | 9.77E-03 |
| **ENSG00000136802** | LRRC8A | 56262 | 1798.72192 | -0.51 | 4.83E-04 | 9.70E-03 |
| **ENSG00000163453** | IGFBP7 | 3490 | 16959.7091 | -0.51 | 8.25E-09 | 6.61E-07 |
| **ENSG00000172270** | BSG | 682 | 2813.11803 | -0.51 | 7.81E-06 | 3.03E-04 |
| **ENSG00000145147** | SLIT2 | 9353 | 1799.96014 | -0.51 | 2.30E-04 | 5.31E-03 |
| **ENSG00000112759** | SLC29A1 | 2030 | 3171.97043 | -0.50 | 1.03E-05 | 3.91E-04 |
| **ENSG00000105755** | ETHE1 | 23474 | 2155.74145 | -0.50 | 2.37E-04 | 5.45E-03 |
| **ENSG00000206560** | ANKRD28 | 23243 | 1888.31872 | -0.50 | 1.78E-04 | 4.28E-03 |
| **ENSG00000103257** | SLC7A5 | 8140 | 6165.66196 | -0.50 | 2.86E-08 | 2.08E-06 |
| **ENSG00000138685** | FGF2 | 2247 | 2979.26582 | -0.50 | 1.28E-05 | 4.71E-04 |
| **ENSG00000113811** | SELENOK | 58515 | 2798.55689 | -0.49 | 4.04E-05 | 1.22E-03 |
| **ENSG00000125484** | GTF3C4 | 9329 | 2009.18701 | -0.49 | 1.40E-04 | 3.53E-03 |
| **ENSG00000095380** | NANS | 54187 | 3866.45576 | -0.49 | 1.73E-06 | 8.20E-05 |
| **ENSG00000089327** | FXYD5 | 53827 | 2063.52198 | -0.49 | 2.50E-04 | 5.71E-03 |
| **ENSG00000136888** | ATP6V1G1 | 9550 | 5564.05225 | -0.48 | 4.66E-07 | 2.61E-05 |
| **ENSG00000105568** | PPP2R1A | 5518 | 2853.57469 | -0.48 | 1.05E-04 | 2.73E-03 |
| **ENSG00000115286** | NDUFS7 | 374291 | 2296.39315 | -0.48 | 2.05E-04 | 4.83E-03 |
| **ENSG00000111961** | SASH1 | 23328 | 3284.94174 | -0.48 | 2.28E-05 | 7.46E-04 |
| **ENSG00000140464** | PML | 5371 | 3305.18063 | -0.47 | 1.51E-05 | 5.37E-04 |
| **ENSG00000073008** | PVR | 5817 | 5966.1959 | -0.46 | 1.16E-06 | 5.85E-05 |
| **ENSG00000141101** | NOB1 | 28987 | 2339.36215 | -0.46 | 2.12E-04 | 4.94E-03 |
| **ENSG00000110090** | CPT1A | 1374 | 3212.2917 | -0.46 | 5.03E-05 | 1.47E-03 |
| **ENSG00000183207** | RUVBL2 | 10856 | 3691.23511 | -0.46 | 2.27E-05 | 7.46E-04 |
| **ENSG00000185483** | ROR1 | 4919 | 2178.79099 | -0.46 | 3.43E-04 | 7.38E-03 |
| **ENSG00000182197** | EXT1 | 2131 | 3796.06897 | -0.46 | 1.04E-04 | 2.71E-03 |
| **ENSG00000105281** | SLC1A5 | 6510 | 5380.19515 | -0.46 | 9.68E-05 | 2.56E-03 |
| **ENSG00000130816** | DNMT1 | 1786 | 9014.33085 | -0.46 | 3.84E-08 | 2.72E-06 |
| **ENSG00000167635** | ZNF146 | 7705 | 3084.43873 | -0.46 | 4.43E-04 | 9.14E-03 |
| **ENSG00000135002** | RFK | 55312 | 2237.78181 | -0.46 | 3.47E-04 | 7.46E-03 |
| **ENSG00000148229** | POLE3 | 54107 | 6541.07426 | -0.45 | 5.12E-07 | 2.83E-05 |
| **ENSG00000176619** | LMNB2 | 84823 | 9440.08574 | -0.45 | 3.51E-08 | 2.53E-06 |
| **ENSG00000105640** | RPL18A | 6142 | 7234.01499 | -0.45 | 6.90E-07 | 3.72E-05 |
| **ENSG00000130255** | RPL36 | 25873 | 37123.1451 | -0.45 | 1.61E-06 | 7.79E-05 |
| **ENSG00000033867** | SLC4A7 | 9497 | 2647.32135 | -0.45 | 1.16E-04 | 2.98E-03 |
| **ENSG00000011451** | WIZ | 58525 | 2079.5368 | -0.45 | 4.98E-04 | 9.96E-03 |
| **ENSG00000120129** | DUSP1 | 1843 | 3905.51605 | -0.45 | 3.22E-05 | 1.01E-03 |
| **ENSG00000076604** | TRAF4 | 9618 | 2330.48637 | -0.44 | 4.46E-04 | 9.19E-03 |
| **ENSG00000122862** | SRGN | 5552 | 7292.41545 | -0.44 | 3.10E-07 | 1.82E-05 |
| **ENSG00000096696** | DSP | 1832 | 6418.80709 | -0.44 | 9.91E-05 | 2.62E-03 |
| **ENSG00000124201** | ZNFX1 | 57169 | 6223.48461 | -0.44 | 2.03E-04 | 4.77E-03 |
| **ENSG00000139211** | AMIGO2 | 347902 | 9585.56875 | -0.44 | 2.90E-07 | 1.71E-05 |
| **ENSG00000149591** | TAGLN | 6876 | 28186.2069 | -0.44 | 6.97E-10 | 6.87E-08 |
| **ENSG00000136942** | RPL35 | 11224 | 26593.3105 | -0.43 | 1.49E-08 | 1.15E-06 |
| **ENSG00000166750** | SLFN5 | 162394 | 2791.25889 | -0.43 | 1.47E-04 | 3.66E-03 |
| **ENSG00000167601** | AXL | 558 | 21998.8693 | -0.43 | 2.72E-06 | 1.21E-04 |
| **ENSG00000114861** | FOXP1 | 27086 | 7213.8387 | -0.43 | 1.40E-06 | 6.85E-05 |
| **ENSG00000168003** | SLC3A2 | 6520 | 5545.45718 | -0.43 | 1.56E-05 | 5.52E-04 |
| **ENSG00000183087** | GAS6 | 2621 | 7863.14743 | -0.43 | 7.02E-07 | 3.77E-05 |
| **ENSG00000076067** | RBMS2 | 5939 | 2322.10876 | -0.43 | 4.77E-04 | 9.60E-03 |
| **ENSG00000162614** | NEXN | 91624 | 3245.73015 | -0.43 | 4.04E-04 | 8.45E-03 |
| **ENSG00000278259** | MYO19 | 80179 | 3010.55923 | -0.43 | 1.02E-04 | 2.66E-03 |
| **ENSG00000174886** | NDUFA11 | 126328 | 9186.69079 | -0.43 | 6.89E-05 | 1.91E-03 |
| **ENSG00000132612** | VPS4A | 27183 | 2996.69559 | -0.42 | 3.69E-04 | 7.87E-03 |
| **ENSG00000179271** | GADD45GIP1 | 90480 | 6104.32404 | -0.42 | 1.47E-05 | 5.24E-04 |
| **ENSG00000119403** | PHF19 | 26147 | 2864.45033 | -0.41 | 4.51E-04 | 9.26E-03 |
| **ENSG00000106723** | SPIN1 | 10927 | 3820.13955 | -0.41 | 7.41E-05 | 2.02E-03 |
| **ENSG00000100911** | PSME2 | 5721 | 4032.07627 | -0.41 | 2.21E-04 | 5.12E-03 |
| **ENSG00000090615** | GOLGA3 | 2802 | 8350.0862 | -0.41 | 2.22E-06 | 1.01E-04 |
| **ENSG00000083845** | RPS5 | 6193 | 41669.5441 | -0.41 | 1.77E-07 | 1.08E-05 |
| **ENSG00000169715** | MT1E | 4493 | 55641.8847 | -0.41 | 5.39E-09 | 4.50E-07 |
| **ENSG00000136068** | FLNB | 2317 | 8376.0888 | -0.41 | 1.63E-06 | 7.86E-05 |
| **ENSG00000156802** | ATAD2 | 29028 | 2903.63173 | -0.41 | 4.72E-04 | 9.53E-03 |
| **ENSG00000196363** | WDR5 | 11091 | 4412.58245 | -0.41 | 1.72E-04 | 4.14E-03 |
| **ENSG00000168374** | ARF4 | 378 | 21512.5167 | -0.41 | 1.83E-08 | 1.38E-06 |
| **ENSG00000071564** | TCF3 | 6929 | 3801.02911 | -0.40 | 1.08E-04 | 2.82E-03 |
| **ENSG00000003989** | SLC7A2 | 6542 | 3574.61892 | -0.40 | 1.91E-04 | 4.54E-03 |
| **ENSG00000178982** | EIF3K | 27335 | 7974.46475 | -0.40 | 2.78E-04 | 6.23E-03 |
| **ENSG00000105372** | RPS19 | 6223 | 85638.584 | -0.40 | 3.17E-07 | 1.84E-05 |
| **ENSG00000150938** | CRIM1 | 51232 | 6855.17893 | -0.40 | 3.07E-05 | 9.69E-04 |
| **ENSG00000179091** | CYC1 | 1537 | 6548.00126 | -0.40 | 6.37E-06 | 2.52E-04 |
| **ENSG00000089220** | PEBP1 | 5037 | 13951.1326 | -0.40 | 1.17E-06 | 5.86E-05 |
| **ENSG00000148248** | SURF4 | 6836 | 13288.3396 | -0.40 | 4.26E-07 | 2.41E-05 |
| **ENSG00000136877** | FPGS | 2356 | 5124.34815 | -0.40 | 5.95E-05 | 1.70E-03 |
| **ENSG00000168175** | MAPK1IP1L | 93487 | 4858.96208 | -0.39 | 8.47E-05 | 2.28E-03 |
| **ENSG00000130311** | DDA1 | 79016 | 4736.90854 | -0.39 | 8.45E-05 | 2.28E-03 |
| **ENSG00000168028** | RPSA | 3921 | 10022.948 | -0.39 | 9.80E-07 | 5.06E-05 |
| **ENSG00000167005** | NUDT21 | 11051 | 4250.89732 | -0.39 | 3.82E-04 | 8.07E-03 |
| **ENSG00000139926** | FRMD6 | 122786 | 3422.04766 | -0.39 | 2.01E-04 | 4.74E-03 |
| **ENSG00000144713** | RPL32 | 6161 | 38636.7131 | -0.39 | 2.49E-07 | 1.49E-05 |
| **ENSG00000055332** | EIF2AK2 | 5610 | 3638.90349 | -0.39 | 4.71E-04 | 9.53E-03 |
| **ENSG00000108107** | RPL28 | 6158 | 15684.5904 | -0.39 | 1.35E-06 | 6.64E-05 |
| **ENSG00000115268** | RPS15 | 6209 | 27331.5211 | -0.38 | 1.08E-06 | 5.49E-05 |
| **ENSG00000153317** | ASAP1 | 50807 | 6979.21601 | -0.38 | 1.56E-05 | 5.52E-04 |
| **ENSG00000099795** | NDUFB7 | 4713 | 9070.23254 | -0.38 | 6.23E-05 | 1.77E-03 |
| **ENSG00000065308** | TRAM2 | 9697 | 9156.68887 | -0.38 | 1.82E-05 | 6.27E-04 |
| **ENSG00000161016** | RPL8 | 6132 | 46455.2471 | -0.38 | 6.28E-07 | 3.43E-05 |
| **ENSG00000058085** | LAMC2 | 3918 | 7549.65253 | -0.38 | 1.37E-05 | 4.95E-04 |
| **ENSG00000188846** | RPL14 | 9045 | 11696.1864 | -0.38 | 6.22E-06 | 2.48E-04 |
| **ENSG00000164741** | DLC1 | 10395 | 10649.5722 | -0.37 | 9.29E-06 | 3.57E-04 |
| **ENSG00000178719** | GRINA | 2907 | 13895.2262 | -0.37 | 2.08E-04 | 4.87E-03 |
| **ENSG00000116679** | IVNS1ABP | 10625 | 3684.29954 | -0.37 | 4.04E-04 | 8.45E-03 |
| **ENSG00000130332** | LSM7 | 51690 | 8005.08326 | -0.37 | 1.18E-04 | 3.02E-03 |
| **ENSG00000187498** | COL4A1 | 1282 | 6213.08159 | -0.37 | 6.40E-05 | 1.80E-03 |
| **ENSG00000163430** | FSTL1 | 11167 | 17309.6749 | -0.37 | 8.67E-07 | 4.55E-05 |
| **ENSG00000261236** | BOP1 | 23246 | 4068.17583 | -0.37 | 2.60E-04 | 5.90E-03 |
| **ENSG00000125753** | VASP | 7408 | 11697.5992 | -0.37 | 2.88E-05 | 9.12E-04 |
| **ENSG00000163171** | CDC42EP3 | 10602 | 5775.05193 | -0.37 | 1.01E-04 | 2.64E-03 |
| **ENSG00000125656** | CLPP | 8192 | 6840.58942 | -0.36 | 1.86E-04 | 4.45E-03 |
| **ENSG00000179051** | RCC2 | 55920 | 18556.0103 | -0.36 | 3.60E-06 | 1.56E-04 |
| **ENSG00000136830** | FAM129B | 64855 | 11263.1708 | -0.36 | 1.20E-05 | 4.45E-04 |
| **ENSG00000137845** | ADAM10 | 102 | 4220.98778 | -0.36 | 4.74E-04 | 9.57E-03 |
| **ENSG00000206503** | HLA-A | 3105 | 22383.2182 | -0.36 | 1.21E-06 | 6.04E-05 |
| **ENSG00000008394** | MGST1 | 4257 | 5057.96534 | -0.36 | 4.33E-04 | 8.97E-03 |
| **ENSG00000074842** | MYDGF | 56005 | 12417.104 | -0.35 | 2.34E-05 | 7.62E-04 |
| **ENSG00000204264** | PSMB8 | 5696 | 5674.81847 | -0.35 | 1.41E-04 | 3.53E-03 |
| **ENSG00000177425** | PAWR | 5074 | 5331.22784 | -0.35 | 3.65E-04 | 7.80E-03 |
| **ENSG00000086065** | CHMP5 | 51510 | 7806.83302 | -0.35 | 1.16E-04 | 2.98E-03 |
| **ENSG00000167110** | GOLGA2 | 2801 | 9575.32588 | -0.35 | 2.09E-05 | 7.03E-04 |
| **ENSG00000107223** | EDF1 | 8721 | 9662.20136 | -0.34 | 1.73E-04 | 4.18E-03 |
| **ENSG00000213024** | NUP62 | 23636 | 5649.44446 | -0.34 | 4.62E-04 | 9.40E-03 |
| **ENSG00000125148** | MT2A | 4502 | 360271.179 | -0.34 | 6.43E-07 | 3.50E-05 |
| **ENSG00000136938** | ANP32B | 10541 | 18245.7823 | -0.34 | 5.50E-06 | 2.23E-04 |
| **ENSG00000126261** | UBA2 | 10054 | 5802.80089 | -0.33 | 2.84E-04 | 6.33E-03 |
| **ENSG00000142534** | RPS11 | 6205 | 37798.9642 | -0.33 | 1.20E-06 | 6.02E-05 |
| **ENSG00000148303** | RPL7A | 6130 | 63141.6527 | -0.33 | 5.78E-06 | 2.32E-04 |
| **ENSG00000067082** | KLF6 | 1316 | 13110.0671 | -0.33 | 1.66E-05 | 5.84E-04 |
| **ENSG00000160209** | PDXK | 8566 | 10897.4739 | -0.33 | 5.08E-05 | 1.48E-03 |
| **ENSG00000011422** | PLAUR | 5329 | 7056.47331 | -0.33 | 1.54E-04 | 3.80E-03 |
| **ENSG00000221983** | UBA52 | 7311 | 30408.7908 | -0.32 | 1.31E-05 | 4.78E-04 |
| **ENSG00000183255** | PTTG1IP | 754 | 10688.7822 | -0.32 | 1.13E-04 | 2.93E-03 |
| **ENSG00000142541** | RPL13A | 23521 | 31185.6815 | -0.31 | 1.03E-05 | 3.91E-04 |
| **ENSG00000063177** | RPL18 | 6141 | 62469.8845 | -0.31 | 1.62E-04 | 3.96E-03 |
| **ENSG00000198242** | RPL23A | 6147 | 70596.4104 | -0.31 | 6.66E-05 | 1.86E-03 |
| **ENSG00000119655** | NPC2 | 10577 | 8165.8048 | -0.30 | 3.21E-04 | 7.03E-03 |
| **ENSG00000188229** | TUBB4B | 10383 | 32508.3584 | -0.29 | 2.52E-05 | 8.13E-04 |
| **ENSG00000198258** | UBL5 | 59286 | 8665.98423 | -0.29 | 3.96E-04 | 8.33E-03 |
| **ENSG00000157020** | SEC13 | 6396 | 9948.53075 | -0.28 | 4.64E-04 | 9.44E-03 |
| **ENSG00000148154** | UGCG | 7357 | 15892.0837 | -0.28 | 2.72E-04 | 6.14E-03 |
| **ENSG00000103187** | COTL1 | 23406 | 33627.1919 | -0.28 | 3.86E-05 | 1.17E-03 |
| **ENSG00000132341** | RAN | 5901 | 44481.5127 | -0.28 | 3.71E-05 | 1.13E-03 |
| **ENSG00000125743** | SNRPD2 | 6633 | 25776.1254 | -0.27 | 4.01E-04 | 8.43E-03 |
| **ENSG00000104529** | EEF1D | 1936 | 21173.2576 | -0.27 | 2.89E-04 | 6.40E-03 |
| **ENSG00000170558** | CDH2 | 1000 | 26904.023 | -0.27 | 2.86E-04 | 6.37E-03 |
| **ENSG00000067560** | RHOA | 387 | 43134.0188 | -0.27 | 1.23E-04 | 3.13E-03 |
| **ENSG00000184640** | SEPT9 | 10801 | 12684.9061 | 0.27 | 4.58E-04 | 9.36E-03 |
| **ENSG00000067225** | PKM | 5315 | 32108.9341 | 0.27 | 1.71E-04 | 4.13E-03 |
| **ENSG00000006451** | RALA | 5898 | 10054.3756 | 0.28 | 4.62E-04 | 9.41E-03 |
| **ENSG00000130508** | PXDN | 7837 | 21034.2529 | 0.28 | 3.72E-04 | 7.89E-03 |
| **ENSG00000157227** | MMP14 | 4323 | 25993.5969 | 0.28 | 6.21E-05 | 1.76E-03 |
| **ENSG00000105968** | H2AFV | 94239 | 17563.088 | 0.30 | 8.21E-05 | 2.23E-03 |
| **ENSG00000164924** | YWHAZ | 7534 | 16033.282 | 0.30 | 3.28E-04 | 7.14E-03 |
| **ENSG00000135624** | CCT7 | 10574 | 11827.309 | 0.31 | 9.07E-05 | 2.42E-03 |
| **ENSG00000166681** | BEX3 | 27018 | 13296.2924 | 0.31 | 1.35E-04 | 3.41E-03 |
| **ENSG00000197746** | PSAP | 5660 | 35127.1112 | 0.31 | 7.56E-06 | 2.95E-04 |
| **ENSG00000111669** | TPI1 | 7167 | 26278.4563 | 0.32 | 9.87E-06 | 3.78E-04 |
| **ENSG00000115484** | CCT4 | 10575 | 14575.8818 | 0.32 | 1.54E-04 | 3.80E-03 |
| **ENSG00000206053** | JPT2 | 90861 | 7372.45992 | 0.32 | 1.84E-04 | 4.39E-03 |
| **ENSG00000143947** | RPS27A | 6233 | 173375.022 | 0.33 | 4.73E-04 | 9.55E-03 |
| **ENSG00000168159** | RNF187 | 149603 | 6964.05997 | 0.33 | 4.13E-04 | 8.61E-03 |
| **ENSG00000137309** | HMGA1 | 3159 | 69775.3272 | 0.33 | 6.54E-06 | 2.58E-04 |
| **ENSG00000094916** | CBX5 | 23468 | 7075.99597 | 0.33 | 2.77E-04 | 6.23E-03 |
| **ENSG00000108100** | CCNY | 219771 | 5061.1308 | 0.34 | 3.67E-04 | 7.84E-03 |
| **ENSG00000148848** | ADAM12 | 8038 | 7252.58508 | 0.34 | 1.59E-04 | 3.89E-03 |
| **ENSG00000149925** | ALDOA | 226 | 27871.8319 | 0.34 | 2.08E-04 | 4.87E-03 |
| **ENSG00000171863** | RPS7 | 6201 | 11990.6243 | 0.35 | 2.88E-05 | 9.12E-04 |
| **ENSG00000100906** | NFKBIA | 4792 | 14144.8662 | 0.35 | 3.40E-05 | 1.06E-03 |
| **ENSG00000171848** | RRM2 | 6241 | 8797.99445 | 0.35 | 2.22E-05 | 7.31E-04 |
| **ENSG00000152104** | PTPN14 | 5784 | 13576.7913 | 0.36 | 2.34E-05 | 7.62E-04 |
| **ENSG00000116260** | QSOX1 | 5768 | 17574.2327 | 0.36 | 1.72E-05 | 6.00E-04 |
| **ENSG00000105835** | NAMPT | 10135 | 5570.57137 | 0.36 | 2.98E-04 | 6.57E-03 |
| **ENSG00000147416** | ATP6V1B2 | 526 | 3716.32252 | 0.36 | 4.81E-04 | 9.68E-03 |
| **ENSG00000113719** | ERGIC1 | 57222 | 10259.5835 | 0.37 | 3.64E-05 | 1.11E-03 |
| **ENSG00000075426** | FOSL2 | 2355 | 5902.79492 | 0.37 | 6.65E-05 | 1.86E-03 |
| **ENSG00000108179** | PPIF | 10105 | 9284.97641 | 0.38 | 3.83E-05 | 1.16E-03 |
| **ENSG00000138069** | RAB1A | 5861 | 4852.01995 | 0.38 | 8.33E-05 | 2.26E-03 |
| **ENSG00000060237** | WNK1 | 65125 | 3909.67368 | 0.38 | 2.13E-04 | 4.96E-03 |
| **ENSG00000145012** | LPP | 4026 | 3387.2004 | 0.39 | 2.96E-04 | 6.54E-03 |
| **ENSG00000154978** | VOPP1 | 81552 | 7837.34676 | 0.39 | 9.91E-06 | 3.79E-04 |
| **ENSG00000115241** | PPM1G | 5496 | 11639.4844 | 0.39 | 1.00E-06 | 5.16E-05 |
| **ENSG00000118816** | CCNI | 10983 | 18199.3873 | 0.39 | 9.87E-08 | 6.38E-06 |
| **ENSG00000118900** | UBN1 | 29855 | 5874.00402 | 0.39 | 3.49E-04 | 7.47E-03 |
| **ENSG00000060982** | BCAT1 | 586 | 8770.69893 | 0.40 | 3.86E-06 | 1.65E-04 |
| **ENSG00000176788** | BASP1 | 10409 | 19493.3789 | 0.40 | 7.92E-08 | 5.25E-06 |
| **ENSG00000222041** | CYTOR | 112597 | 4307.94357 | 0.40 | 6.83E-05 | 1.90E-03 |
| **ENSG00000173706** | HEG1 | 57493 | 5325.65094 | 0.40 | 1.54E-05 | 5.45E-04 |
| **ENSG00000053371** | AKR7A2 | 8574 | 3952.20259 | 0.40 | 1.45E-04 | 3.62E-03 |
| **ENSG00000127952** | STYXL1 | 51657 | 3313.38468 | 0.40 | 1.75E-04 | 4.19E-03 |
| **ENSG00000204843** | DCTN1 | 1639 | 3577.43738 | 0.40 | 1.68E-04 | 4.08E-03 |
| **ENSG00000162063** | CCNF | 899 | 3275.1009 | 0.41 | 2.37E-04 | 5.45E-03 |
| **ENSG00000147454** | SLC25A37 | 51312 | 8825.50379 | 0.41 | 9.55E-07 | 4.96E-05 |
| **ENSG00000134802** | SLC43A3 | 29015 | 4045.24623 | 0.41 | 5.46E-05 | 1.58E-03 |
| **ENSG00000135842** | FAM129A | 116496 | 4599.79736 | 0.42 | 2.35E-05 | 7.64E-04 |
| **ENSG00000162493** | PDPN | 10630 | 5007.93906 | 0.42 | 1.96E-05 | 6.63E-04 |
| **ENSG00000253368** | TRNP1 | 388610 | 4631.00057 | 0.42 | 3.38E-04 | 7.31E-03 |
| **ENSG00000197157** | SND1 | 27044 | 7042.43697 | 0.42 | 2.40E-05 | 7.80E-04 |
| **ENSG00000072682** | P4HA2 | 8974 | 7218.22322 | 0.42 | 1.46E-06 | 7.10E-05 |
| **ENSG00000034510** | TMSB10 | 9168 | 54797.9352 | 0.42 | 2.54E-09 | 2.24E-07 |
| **ENSG00000166949** | SMAD3 | 4088 | 5968.88168 | 0.43 | 1.47E-05 | 5.24E-04 |
| **ENSG00000013364** | MVP | 9961 | 3128.35372 | 0.43 | 1.92E-04 | 4.54E-03 |
| **ENSG00000115561** | CHMP3 | 51652 | 6645.62767 | 0.43 | 1.70E-05 | 5.93E-04 |
| **ENSG00000026508** | CD44 | 960 | 26807.435 | 0.43 | 3.62E-09 | 3.11E-07 |
| **ENSG00000167323** | STIM1 | 6786 | 3575.92888 | 0.43 | 6.17E-05 | 1.75E-03 |
| **ENSG00000102034** | ELF4 | 2000 | 2720.47351 | 0.44 | 2.84E-04 | 6.33E-03 |
| **ENSG00000182287** | AP1S2 | 8905 | 3047.7883 | 0.44 | 9.99E-05 | 2.63E-03 |
| **ENSG00000140990** | NDUFB10 | 4716 | 5451.79385 | 0.44 | 1.10E-05 | 4.13E-04 |
| **ENSG00000058056** | USP13 | 8975 | 2902.52791 | 0.44 | 9.13E-05 | 2.44E-03 |
| ENSG00000126005 | |  | 3360.10913 | 0.45 | 2.60E-05 | 8.37E-04 |
| **ENSG00000071242** | RPS6KA2 | 6196 | 5807.64905 | 0.45 | 1.01E-05 | 3.84E-04 |
| **ENSG00000068489** | PRR11 | 55771 | 10471.4867 | 0.45 | 2.01E-04 | 4.74E-03 |
| **ENSG00000151892** | GFRA1 | 2674 | 5761.83784 | 0.45 | 1.24E-05 | 4.58E-04 |
| **ENSG00000169592** | INO80E | 283899 | 2594.7095 | 0.45 | 3.29E-04 | 7.15E-03 |
| **ENSG00000129680** | MAP7D3 | 79649 | 2401.2636 | 0.45 | 3.77E-04 | 7.99E-03 |
| **ENSG00000244509** | APOBEC3C | 27350 | 2326.85856 | 0.46 | 2.58E-04 | 5.88E-03 |
| **ENSG00000176171** | BNIP3 | 664 | 3896.13689 | 0.46 | 1.49E-04 | 3.71E-03 |
| **ENSG00000112697** | TMEM30A | 55754 | 5928.96157 | 0.46 | 3.80E-06 | 1.64E-04 |
| **ENSG00000172216** | CEBPB | 1051 | 3781.17868 | 0.46 | 7.74E-06 | 3.02E-04 |
| **ENSG00000198959** | TGM2 | 7052 | 5447.11546 | 0.46 | 4.95E-06 | 2.03E-04 |
| **ENSG00000173327** | MAP3K11 | 4296 | 2144.54531 | 0.46 | 3.38E-04 | 7.31E-03 |
| **ENSG00000180398** | MCFD2 | 90411 | 2636.12665 | 0.46 | 1.19E-04 | 3.04E-03 |
| **ENSG00000070614** | NDST1 | 3340 | 11758.4096 | 0.47 | 7.24E-07 | 3.87E-05 |
| **ENSG00000096433** | ITPR3 | 3710 | 3610.38111 | 0.47 | 3.18E-05 | 9.97E-04 |
| **ENSG00000138071** | ACTR2 | 10097 | 9821.33648 | 0.48 | 4.24E-09 | 3.63E-07 |
| **ENSG00000077235** | GTF3C1 | 2975 | 2650.97495 | 0.48 | 2.78E-04 | 6.23E-03 |
| **ENSG00000103202** | NME4 | 4833 | 3130.59599 | 0.48 | 7.28E-05 | 2.00E-03 |
| **ENSG00000103266** | STUB1 | 10273 | 4599.76144 | 0.48 | 1.95E-06 | 9.06E-05 |
| **ENSG00000102312** | PORCN | 64840 | 2205.76334 | 0.48 | 1.29E-04 | 3.28E-03 |
| **ENSG00000168209** | DDIT4 | 54541 | 5363.09695 | 0.48 | 4.11E-07 | 2.33E-05 |
| **ENSG00000139083** | ETV6 | 2120 | 2354.6039 | 0.49 | 7.37E-05 | 2.01E-03 |
| **ENSG00000100354** | TNRC6B | 23112 | 2190.76053 | 0.49 | 3.84E-04 | 8.10E-03 |
| **ENSG00000100647** | SUSD6 | 9766 | 2515.38949 | 0.49 | 1.57E-04 | 3.86E-03 |
| **ENSG00000115306** | SPTBN1 | 6711 | 20244.2251 | 0.49 | 9.24E-12 | 1.10E-09 |
| **ENSG00000141526** | SLC16A3 | 9123 | 20172.9357 | 0.49 | 7.07E-09 | 5.75E-07 |
| **ENSG00000115216** | NRBP1 | 29959 | 7814.72762 | 0.49 | 8.43E-08 | 5.56E-06 |
| **ENSG00000035862** | TIMP2 | 7077 | 9658.63948 | 0.49 | 9.18E-10 | 8.83E-08 |
| **ENSG00000151694** | ADAM17 | 6868 | 3726.75643 | 0.49 | 3.85E-05 | 1.16E-03 |
| **ENSG00000166741** | NNMT | 4837 | 25283.5201 | 0.50 | 3.07E-09 | 2.68E-07 |
| **ENSG00000149485** | FADS1 | 3992 | 5251.20253 | 0.50 | 2.91E-05 | 9.18E-04 |
| **ENSG00000153823** | PID1 | 55022 | 1834.87681 | 0.50 | 3.79E-04 | 8.02E-03 |
| **ENSG00000171792** | RHNO1 | 83695 | 1584.15192 | 0.50 | 4.54E-04 | 9.30E-03 |
| **ENSG00000011028** | MRC2 | 9902 | 3564.12847 | 0.50 | 3.61E-05 | 1.11E-03 |
| ENSG00000254615 | |  | 1757.42631 | 0.50 | 2.75E-04 | 6.20E-03 |
| **ENSG00000148908** | RGS10 | 6001 | 2352.57777 | 0.51 | 1.16E-04 | 2.99E-03 |
| **ENSG00000198522** | GPN1 | 11321 | 2355.11218 | 0.51 | 4.66E-05 | 1.37E-03 |
| **ENSG00000221823** | PPP3R1 | 5534 | 2558.8253 | 0.51 | 2.48E-04 | 5.68E-03 |
| **ENSG00000157045** | NTAN1 | 123803 | 4584.19847 | 0.51 | 2.09E-06 | 9.59E-05 |
| **ENSG00000111817** | DSE | 29940 | 2190.55951 | 0.51 | 1.01E-04 | 2.64E-03 |
| **ENSG00000126351** | THRA | 7067 | 1430.51184 | 0.51 | 4.41E-04 | 9.11E-03 |
| **ENSG00000106266** | SNX8 | 29886 | 2022.80855 | 0.51 | 5.69E-05 | 1.64E-03 |
| **ENSG00000168894** | RNF181 | 51255 | 6791.80656 | 0.52 | 3.96E-08 | 2.77E-06 |
| **ENSG00000173210** | ABLIM3 | 22885 | 2140.61554 | 0.52 | 4.27E-05 | 1.27E-03 |
| **ENSG00000112893** | MAN2A1 | 4124 | 4088.21644 | 0.52 | 2.26E-06 | 1.03E-04 |
| **ENSG00000072274** | TFRC | 7037 | 5446.54238 | 0.52 | 4.01E-07 | 2.29E-05 |
| **ENSG00000204568** | MRPS18B | 28973 | 12266.699 | 0.53 | 4.78E-08 | 3.31E-06 |
| **ENSG00000170525** | PFKFB3 | 5209 | 2426.76037 | 0.53 | 7.18E-05 | 1.98E-03 |
| **ENSG00000028277** | POU2F2 | 5452 | 2539.36281 | 0.53 | 1.31E-05 | 4.79E-04 |
| **ENSG00000143870** | PDIA6 | 10130 | 16805.097 | 0.53 | 8.96E-13 | 1.17E-10 |
| **ENSG00000171729** | TMEM51 | 55092 | 3075.80504 | 0.53 | 5.76E-06 | 2.32E-04 |
| **ENSG00000042493** | CAPG | 822 | 5085.104 | 0.53 | 2.55E-08 | 1.88E-06 |
| **ENSG00000110066** | KMT5B | 51111 | 1522.41002 | 0.53 | 4.60E-04 | 9.37E-03 |
| **ENSG00000115234** | SNX17 | 9784 | 3337.44467 | 0.53 | 5.77E-06 | 2.32E-04 |
| **ENSG00000136717** | BIN1 | 274 | 3159.699 | 0.53 | 1.28E-06 | 6.32E-05 |
| **ENSG00000101079** | NDRG3 | 57446 | 2027.655 | 0.54 | 1.56E-04 | 3.85E-03 |
| **ENSG00000124145** | SDC4 | 6385 | 28515.3266 | 0.54 | 1.96E-14 | 3.09E-12 |
| **ENSG00000143924** | EML4 | 27436 | 3060.94308 | 0.54 | 5.39E-06 | 2.20E-04 |
| **ENSG00000124356** | STAMBP | 10617 | 2565.57031 | 0.54 | 4.53E-06 | 1.90E-04 |
| **ENSG00000145779** | TNFAIP8 | 25816 | 2286.75579 | 0.54 | 2.77E-05 | 8.84E-04 |
| **ENSG00000118257** | NRP2 | 8828 | 1867.53396 | 0.54 | 4.30E-05 | 1.28E-03 |
| **ENSG00000167996** | FTH1 | 2495 | 59280.9867 | 0.55 | 2.36E-14 | 3.67E-12 |
| **ENSG00000143786** | CNIH3 | 149111 | 1336.6438 | 0.55 | 4.48E-04 | 9.22E-03 |
| **ENSG00000103876** | FAH | 2184 | 1828.66794 | 0.55 | 2.20E-04 | 5.11E-03 |
| **ENSG00000050165** | 3 DKK | 27122 | 8522.79101 | 0.55 | 1.91E-11 | 2.23E-09 |
| **ENSG00000163798** | SLC4A1AP | 22950 | 1804.20167 | 0.55 | 5.83E-05 | 1.67E-03 |
| **ENSG00000101040** | ZMYND8 | 23613 | 2379.29556 | 0.56 | 1.41E-05 | 5.07E-04 |
| **ENSG00000171914** | TLN2 | 83660 | 1165.01741 | 0.57 | 4.34E-04 | 8.97E-03 |
| **ENSG00000145901** | TNIP1 | 10318 | 14926.8652 | 0.57 | 2.02E-11 | 2.35E-09 |
| **ENSG00000103496** | STX4 | 6810 | 2455.24672 | 0.57 | 2.11E-06 | 9.66E-05 |
| **ENSG00000175352** | NRIP3 | 56675 | 3269.09292 | 0.57 | 1.84E-06 | 8.63E-05 |
| **ENSG00000134308** | YWHAQ | 10971 | 7175.49494 | 0.57 | 5.21E-09 | 4.37E-07 |
| **ENSG00000135245** | HILPDA | 29923 | 2128.77709 | 0.57 | 5.48E-05 | 1.58E-03 |
| **ENSG00000104635** | SLC39A14 | 23516 | 3579.31269 | 0.57 | 6.96E-07 | 3.75E-05 |
| **ENSG00000103415** | HMOX2 | 3163 | 1405.89277 | 0.57 | 3.36E-04 | 7.29E-03 |
| **ENSG00000166033** | HTRA1 | 5654 | 2916.06509 | 0.57 | 2.52E-06 | 1.13E-04 |
| **ENSG00000134318** | ROCK2 | 9475 | 2762.76291 | 0.57 | 4.05E-05 | 1.22E-03 |
| **ENSG00000127920** | GNG11 | 2791 | 2608.30778 | 0.58 | 1.72E-05 | 6.00E-04 |
| **ENSG00000104412** | EMC2 | 9694 | 2515.11735 | 0.58 | 5.16E-06 | 2.12E-04 |
| **ENSG00000145979** | TBC1D7 | 51256 | 1612.08134 | 0.58 | 5.39E-05 | 1.56E-03 |
| **ENSG00000124788** | ATXN1 | 6310 | 1215.81952 | 0.59 | 4.49E-04 | 9.22E-03 |
| **ENSG00000101160** | CTSZ | 1522 | 5207.48792 | 0.59 | 7.81E-09 | 6.29E-07 |
| **ENSG00000176407** | KCMF1 | 56888 | 2468.12772 | 0.59 | 1.04E-05 | 3.93E-04 |
| **ENSG00000099194** | SCD | 6319 | 1481.28194 | 0.59 | 9.22E-05 | 2.46E-03 |
| **ENSG00000102882** | MAPK3 | 5595 | 2464.14839 | 0.59 | 4.66E-06 | 1.94E-04 |
| **ENSG00000104765** | BNIP3L | 665 | 2043.80382 | 0.59 | 6.32E-06 | 2.51E-04 |
| **ENSG00000152465** | NMT2 | 9397 | 2579.9199 | 0.59 | 1.37E-05 | 4.95E-04 |
| **ENSG00000213639** | PPP1CB | 5500 | 4926.18788 | 0.60 | 3.33E-09 | 2.90E-07 |
| **ENSG00000114956** | DGUOK | 1716 | 3111.22701 | 0.60 | 8.99E-07 | 4.70E-05 |
| **ENSG00000245532** | NEAT1 | 283131 | 5299.09132 | 0.60 | 3.58E-09 | 3.10E-07 |
| **ENSG00000138386** | NAB1 | 4664 | 1299.47952 | 0.60 | 1.19E-04 | 3.04E-03 |
| **ENSG00000120533** | ENY2 | 56943 | 8857.54571 | 0.60 | 1.02E-09 | 9.64E-08 |
| **ENSG00000147536** | GINS4 | 84296 | 1113.12404 | 0.60 | 2.47E-04 | 5.65E-03 |
| **ENSG00000186480** | INSIG1 | 3638 | 1629.00954 | 0.61 | 5.79E-05 | 1.66E-03 |
| **ENSG00000134330** | IAH1 | 285148 | 1658.18595 | 0.61 | 1.34E-05 | 4.88E-04 |
| **ENSG00000100503** | NIN | 51199 | 1822.80797 | 0.61 | 6.01E-06 | 2.41E-04 |
| **ENSG00000031081** | ARHGAP31 | 57514 | 1112.42559 | 0.61 | 3.27E-04 | 7.13E-03 |
| **ENSG00000106003** | LFNG | 3955 | 1345.5999 | 0.61 | 1.62E-04 | 3.96E-03 |
| **ENSG00000115652** | UXS1 | 80146 | 4950.5014 | 0.62 | 1.52E-09 | 1.40E-07 |
| **ENSG00000164292** | RHOBTB3 | 22836 | 10993.2852 | 0.62 | 6.83E-14 | 1.03E-11 |
| **ENSG00000118096** | IFT46 | 56912 | 967.604533 | 0.62 | 3.31E-04 | 7.18E-03 |
| **ENSG00000115355** | CCDC88A | 55704 | 1598.05543 | 0.63 | 1.85E-05 | 6.36E-04 |
| **ENSG00000198324** | FAM109A | 144717 | 1024.3037 | 0.63 | 2.92E-04 | 6.47E-03 |
| **ENSG00000068878** | PSME4 | 23198 | 2955.01871 | 0.63 | 4.69E-07 | 2.62E-05 |
| **ENSG00000122861** | PLAU | 5328 | 4606.17234 | 0.63 | 1.05E-05 | 3.94E-04 |
| **ENSG00000153406** | NMRAL1 | 57407 | 1389.64447 | 0.63 | 1.78E-05 | 6.17E-04 |
| **ENSG00000125872** | LRRN4 | 164312 | 1312.29154 | 0.64 | 3.23E-05 | 1.01E-03 |
| **ENSG00000163734** | CXCL3 | 2921 | 2966.899 | 0.65 | 3.17E-07 | 1.84E-05 |
| **ENSG00000057935** | MTA3 | 57504 | 1073.86115 | 0.65 | 1.69E-04 | 4.10E-03 |
| **ENSG00000072864** | NDE1 | 54820 | 1402.96539 | 0.66 | 2.20E-05 | 7.28E-04 |
| **ENSG00000115977** | AAK1 | 22848 | 2898.61736 | 0.66 | 1.82E-08 | 1.38E-06 |
| **ENSG00000111674** | ENO2 | 2026 | 1510.97921 | 0.66 | 4.69E-06 | 1.95E-04 |
| **ENSG00000085662** | AKR1B1 | 231 | 27711.9155 | 0.66 | 4.42E-20 | 1.25E-17 |
| **ENSG00000166592** | RRAD | 6236 | 1736.32281 | 0.66 | 1.24E-05 | 4.58E-04 |
| **ENSG00000011105** | TSPAN9 | 10867 | 1464.93734 | 0.66 | 1.21E-04 | 3.09E-03 |
| **ENSG00000144802** | NFKBIZ | 64332 | 3382.21862 | 0.66 | 1.25E-09 | 1.16E-07 |
| **ENSG00000152291** | TGOLN2 | 10618 | 8593.75209 | 0.67 | 1.33E-08 | 1.03E-06 |
| **ENSG00000182534** | MXRA7 | 439921 | 3364.31751 | 0.67 | 4.44E-10 | 4.46E-08 |
| **ENSG00000101400** | SNTA1 | 6640 | 1987.43497 | 0.67 | 2.70E-07 | 1.61E-05 |
| **ENSG00000197321** | SVIL | 6840 | 1525.79361 | 0.68 | 1.80E-06 | 8.47E-05 |
| **ENSG00000137393** | RNF144B | 255488 | 1731.22843 | 0.68 | 4.20E-06 | 1.78E-04 |
| **ENSG00000108960** | MMD | 23531 | 962.265271 | 0.69 | 2.60E-04 | 5.90E-03 |
| **ENSG00000143797** | MBOAT2 | 129642 | 853.537413 | 0.69 | 1.88E-04 | 4.49E-03 |
| **ENSG00000074410** | CA12 | 771 | 2947.47805 | 0.69 | 6.43E-08 | 4.33E-06 |
| **ENSG00000168883** | USP39 | 10713 | 1684.97863 | 0.69 | 1.70E-06 | 8.10E-05 |
| **ENSG00000109854** | HTATIP2 | 10553 | 1178.21135 | 0.69 | 3.26E-05 | 1.01E-03 |
| **ENSG00000112715** | VEGFA | 7422 | 2736.27098 | 0.70 | 5.17E-09 | 4.36E-07 |
| **ENSG00000103148** | NPRL3 | 8131 | 1078.1213 | 0.70 | 3.72E-05 | 1.13E-03 |
| **ENSG00000125618** | PAX8 | 7849 | 11825.4227 | 0.70 | 6.09E-19 | 1.58E-16 |
| **ENSG00000176845** | METRNL | 284207 | 891.455556 | 0.70 | 1.41E-04 | 3.53E-03 |
| **ENSG00000186918** | ZNF395 | 55893 | 1455.90126 | 0.70 | 1.31E-06 | 6.47E-05 |
| **ENSG00000005448** | WDR54 | 84058 | 2391.62308 | 0.70 | 1.14E-07 | 7.28E-06 |
| **ENSG00000115756** | HPCAL1 | 3241 | 2521.00531 | 0.71 | 9.46E-09 | 7.51E-07 |
| **ENSG00000081041** | CXCL2 | 2920 | 2710.54569 | 0.71 | 3.29E-07 | 1.90E-05 |
| **ENSG00000147654** | EBAG9 | 9166 | 1116.90266 | 0.71 | 5.39E-05 | 1.56E-03 |
| **ENSG00000092607** | TBX15 | 6913 | 1073.91382 | 0.71 | 4.65E-05 | 1.37E-03 |
| **ENSG00000204536** | CCHCR1 | 54535 | 1355.26064 | 0.71 | 6.68E-05 | 1.87E-03 |
| **ENSG00000105825** | TFPI2 | 7980 | 5636.19303 | 0.72 | 1.02E-13 | 1.49E-11 |
| **ENSG00000167191** | GPRC5B | 51704 | 1138.08086 | 0.73 | 3.54E-05 | 1.09E-03 |
| **ENSG00000174791** | RIN1 | 9610 | 723.143571 | 0.73 | 2.37E-04 | 5.45E-03 |
| **ENSG00000163735** | CXCL5 | 6374 | 58285.5349 | 0.73 | 1.31E-24 | 4.77E-22 |
| **ENSG00000171604** | CXXC5 | 51523 | 3257.27057 | 0.74 | 1.62E-10 | 1.70E-08 |
| **ENSG00000164932** | CTHRC1 | 115908 | 1728.38318 | 0.75 | 1.09E-07 | 7.05E-06 |
| **ENSG00000101017** | CD40 | 958 | 1658.38935 | 0.75 | 7.65E-07 | 4.05E-05 |
| **ENSG00000106330** | MOSPD3 | 64598 | 837.390098 | 0.75 | 5.36E-05 | 1.56E-03 |
| **ENSG00000128641** | MYO1B | 4430 | 5555.04749 | 0.75 | 1.24E-12 | 1.60E-10 |
| **ENSG00000115009** | CCL20 | 6364 | 936.93104 | 0.76 | 4.74E-05 | 1.39E-03 |
| **ENSG00000104408** | EIF3E | 3646 | 2952.84851 | 0.77 | 9.18E-11 | 1.00E-08 |
| **ENSG00000253522** | MIR3142HG | 107075116 | 787.995753 | 0.77 | 9.99E-05 | 2.63E-03 |
| **ENSG00000171608** | PIK3CD | 5293 | 3619.48801 | 0.77 | 7.36E-10 | 7.12E-08 |
| **ENSG00000113140** | SPARC | 6678 | 17283.9504 | 0.78 | 8.24E-26 | 3.21E-23 |
| **ENSG00000159164** | SV2A | 9900 | 747.542381 | 0.78 | 9.07E-05 | 2.42E-03 |
| **ENSG00000153048** | CARHSP1 | 23589 | 3976.18846 | 0.78 | 1.30E-14 | 2.18E-12 |
| **ENSG00000134668** | SPOCD1 | 90853 | 1246.15964 | 0.78 | 5.23E-07 | 2.88E-05 |
| **ENSG00000157514** | TSC22D3 | 1831 | 1825.00769 | 0.79 | 4.37E-06 | 1.84E-04 |
| **ENSG00000169515** | CCDC8 | 83987 | 788.193515 | 0.79 | 3.58E-05 | 1.10E-03 |
| **ENSG00000212747** | RTL8B | 441518 | 615.031904 | 0.80 | 1.90E-04 | 4.51E-03 |
| **ENSG00000124875** | CXCL6 | 6372 | 18934.5299 | 0.80 | 2.28E-15 | 4.05E-13 |
| **ENSG00000184232** | OAF | 220323 | 984.159706 | 0.80 | 5.51E-06 | 2.23E-04 |
| **ENSG00000166250** | CLMP | 79827 | 1829.96754 | 0.81 | 2.96E-09 | 2.60E-07 |
| **ENSG00000189067** | LITAF | 9516 | 2520.68479 | 0.82 | 4.06E-11 | 4.58E-09 |
| **ENSG00000116005** | PCYOX1 | 51449 | 1516.57385 | 0.82 | 3.69E-08 | 2.63E-06 |
| **ENSG00000131435** | PDLIM4 | 8572 | 8246.63365 | 0.82 | 3.09E-17 | 6.75E-15 |
| **ENSG00000010818** | HIVEP2 | 3097 | 1614.32271 | 0.83 | 1.61E-08 | 1.24E-06 |
| **ENSG00000111799** | COL12A1 | 1303 | 974.229206 | 0.83 | 1.12E-04 | 2.91E-03 |
| **ENSG00000100092** | SH3BP1 | 23616 | 1342.99272 | 0.84 | 7.69E-07 | 4.06E-05 |
| **ENSG00000163874** | ZC3H12A | 80149 | 4497.7783 | 0.85 | 2.79E-16 | 5.30E-14 |
| **ENSG00000123096** | SSPN | 8082 | 639.719579 | 0.85 | 9.34E-05 | 2.48E-03 |
| **ENSG00000166922** | SCG5 | 6447 | 681.386843 | 0.86 | 1.90E-05 | 6.49E-04 |
| **ENSG00000112096** | SOD2 | 6648 | 10753.5748 | 0.87 | 2.01E-17 | 4.56E-15 |
| **ENSG00000197872** | FAM49A | 81553 | 837.432712 | 0.87 | 5.24E-06 | 2.14E-04 |
| **ENSG00000088826** | SMOX | 54498 | 1558.83914 | 0.87 | 3.69E-08 | 2.63E-06 |
| **ENSG00000135678** | CPM | 1368 | 1769.98512 | 0.87 | 1.69E-06 | 8.08E-05 |
| **ENSG00000127946** | HIP1 | 3092 | 851.192415 | 0.88 | 6.31E-06 | 2.51E-04 |
| **ENSG00000163739** | CXCL1 | 2919 | 94984.792 | 0.88 | 2.11E-40 | 1.50E-37 |
| **ENSG00000089723** | OTUB2 | 78990 | 771.910135 | 0.89 | 3.74E-06 | 1.62E-04 |
| **ENSG00000089486** | CDIP1 | 29965 | 465.191946 | 0.89 | 4.16E-04 | 8.65E-03 |
| **ENSG00000154928** | EPHB1 | 2047 | 485.93237 | 0.89 | 3.83E-04 | 8.08E-03 |
| **ENSG00000156011** | PSD3 | 23362 | 1558.512 | 0.89 | 1.23E-09 | 1.15E-07 |
| **ENSG00000124813** | RUNX2 | 860 | 2469.23604 | 0.90 | 3.87E-10 | 3.91E-08 |
| **ENSG00000159167** | STC1 | 6781 | 1467.21936 | 0.90 | 1.25E-08 | 9.86E-07 |
| **ENSG00000115648** | MLPH | 79083 | 1227.25653 | 0.91 | 8.04E-07 | 4.23E-05 |
| **ENSG00000057704** | TMCC3 | 57458 | 962.380075 | 0.91 | 1.95E-05 | 6.63E-04 |
| **ENSG00000090376** | IRAK3 | 11213 | 905.034869 | 0.91 | 7.85E-06 | 3.04E-04 |
| **ENSG00000160991** | ORAI2 | 80228 | 2036.29396 | 0.92 | 4.90E-08 | 3.38E-06 |
| **ENSG00000139629** | GALNT6 | 11226 | 1541.86669 | 0.93 | 7.47E-06 | 2.92E-04 |
| **ENSG00000186815** | TPCN1 | 53373 | 1605.40565 | 0.93 | 2.42E-08 | 1.80E-06 |
| **ENSG00000115884** | SDC1 | 6382 | 3231.06688 | 0.93 | 5.96E-17 | 1.20E-14 |
| **ENSG00000123095** | BHLHE41 | 79365 | 856.583984 | 0.93 | 3.15E-07 | 1.84E-05 |
| **ENSG00000138061** | CYP1B1 | 1545 | 742.970134 | 0.94 | 3.97E-06 | 1.70E-04 |
| **ENSG00000134202** | GSTM3 | 2947 | 561.935744 | 0.94 | 2.71E-05 | 8.67E-04 |
| **ENSG00000172731** | LRRC20 | 55222 | 388.865934 | 0.95 | 3.01E-04 | 6.64E-03 |
| **ENSG00000128340** | RAC2 | 5880 | 2292.35565 | 0.96 | 4.28E-14 | 6.54E-12 |
| **ENSG00000180287** | PLD5 | 200150 | 1286.06374 | 0.96 | 5.10E-09 | 4.32E-07 |
| **ENSG00000144834** | TAGLN3 | 29114 | 385.136626 | 0.96 | 2.50E-04 | 5.71E-03 |
| **ENSG00000173442** | EHBP1L1 | 254102 | 1245.88806 | 0.98 | 7.24E-09 | 5.86E-07 |
| **ENSG00000113319** | RASGRF2 | 5924 | 464.087984 | 0.99 | 1.44E-04 | 3.61E-03 |
| **ENSG00000168528** | SERINC2 | 347735 | 2907.20134 | 1.00 | 1.08E-18 | 2.71E-16 |
| **ENSG00000156711** | MAPK13 | 5603 | 328.872034 | 1.00 | 4.56E-04 | 9.33E-03 |
| **ENSG00000196154** | S100A4 | 6275 | 418.207392 | 1.00 | 7.04E-05 | 1.95E-03 |
| **ENSG00000122729** | ACO1 | 48 | 4049.86179 | 1.00 | 7.19E-20 | 1.97E-17 |
| **ENSG00000166523** | CLEC4E | 26253 | 450.052141 | 1.03 | 2.16E-05 | 7.21E-04 |
| **ENSG00000166920** | C15orf48 | 84419 | 624.057803 | 1.04 | 1.46E-06 | 7.09E-05 |
| **ENSG00000120549** | KIAA1217 | 56243 | 659.189218 | 1.04 | 6.30E-07 | 3.43E-05 |
| **ENSG00000168874** | ATOH8 | 84913 | 436.094044 | 1.04 | 8.02E-05 | 2.18E-03 |
| **ENSG00000163637** | PRICKLE2 | 166336 | 832.647548 | 1.04 | 1.62E-08 | 1.24E-06 |
| **ENSG00000085117** | CD82 | 3732 | 6697.58375 | 1.05 | 1.08E-18 | 2.71E-16 |
| **ENSG00000177706** | FAM20C | 56975 | 2982.86622 | 1.05 | 5.70E-17 | 1.17E-14 |
| **ENSG00000103485** | QPRT | 23475 | 6047.47278 | 1.06 | 1.89E-32 | 9.98E-30 |
| **ENSG00000142173** | COL6A2 | 1292 | 1292.89774 | 1.07 | 2.17E-10 | 2.26E-08 |
| **ENSG00000154134** | ROBO3 | 64221 | 660.109221 | 1.08 | 4.77E-07 | 2.65E-05 |
| **ENSG00000124126** | PREX1 | 57580 | 703.200713 | 1.09 | 2.61E-06 | 1.17E-04 |
| **ENSG00000127399** | LRRC61 | 65999 | 335.111128 | 1.09 | 1.65E-04 | 4.01E-03 |
| **ENSG00000111275** | ALDH2 | 217 | 373.83021 | 1.09 | 4.52E-05 | 1.33E-03 |
| **ENSG00000118785** | SPP1 | 6696 | 494.660385 | 1.09 | 4.85E-06 | 2.00E-04 |
| **ENSG00000137491** | SLCO2B1 | 11309 | 554.279767 | 1.10 | 1.08E-06 | 5.49E-05 |
| **ENSG00000154146** | NRGN | 4900 | 529.426184 | 1.11 | 4.45E-06 | 1.87E-04 |
| **ENSG00000167105** | TMEM92 | 162461 | 400.599599 | 1.11 | 1.67E-05 | 5.86E-04 |
| **ENSG00000079215** | SLC1A3 | 6507 | 529.377 | 1.12 | 1.79E-06 | 8.46E-05 |
| **ENSG00000065989** | PDE4A | 5141 | 895.97942 | 1.12 | 4.70E-08 | 3.27E-06 |
| **ENSG00000168899** | VAMP5 | 10791 | 368.119288 | 1.13 | 3.59E-05 | 1.10E-03 |
| **ENSG00000151136** | BTBD11 | 121551 | 593.514438 | 1.13 | 3.46E-07 | 1.99E-05 |
| **ENSG00000109107** | ALDOC | 230 | 284.253536 | 1.14 | 1.97E-04 | 4.66E-03 |
| **ENSG00000125538** | IL1B | 3553 | 640.850045 | 1.15 | 2.52E-07 | 1.50E-05 |
| **ENSG00000103260** | METRN | 79006 | 382.529536 | 1.15 | 2.11E-05 | 7.07E-04 |
| **ENSG00000115594** | IL1R1 | 3554 | 440.03904 | 1.16 | 3.48E-05 | 1.08E-03 |
| **ENSG00000114251** | WNT5A | 7474 | 2724.22192 | 1.16 | 7.19E-24 | 2.50E-21 |
| **ENSG00000084693** | AGBL5 | 60509 | 501.210615 | 1.17 | 1.42E-05 | 5.10E-04 |
| **ENSG00000087245** | MMP2 | 4313 | 436.84168 | 1.17 | 4.71E-06 | 1.95E-04 |
| **ENSG00000142156** | COL6A1 | 1291 | 1726.77022 | 1.18 | 4.45E-18 | 1.05E-15 |
| **ENSG00000171813** | PWWP2B | 170394 | 252.20506 | 1.19 | 2.61E-04 | 5.92E-03 |
| **ENSG00000133805** | AMPD3 | 272 | 485.267392 | 1.21 | 1.36E-06 | 6.67E-05 |
| **ENSG00000125170** | DOK4 | 55715 | 627.210339 | 1.21 | 7.73E-08 | 5.16E-06 |
| **ENSG00000170412** | GPRC5C | 55890 | 1933.94257 | 1.23 | 1.48E-15 | 2.69E-13 |
| **ENSG00000141068** | KSR1 | 8844 | 293.036621 | 1.25 | 5.34E-05 | 1.55E-03 |
| **ENSG00000132429** | POPDC3 | 64208 | 405.791336 | 1.28 | 3.11E-05 | 9.77E-04 |
| ENSG00000236453 | |  | 397.515184 | 1.28 | 2.05E-06 | 9.48E-05 |
| **ENSG00000073150** | PANX2 | 56666 | 282.547929 | 1.32 | 1.87E-05 | 6.42E-04 |
| **ENSG00000173918** | C1QTNF1 | 114897 | 1006.48629 | 1.33 | 2.04E-12 | 2.57E-10 |
| ENSG00000272831 | |  | 199.786887 | 1.33 | 2.66E-04 | 6.01E-03 |
| **ENSG00000119698** | PPP4R4 | 57718 | 178.847986 | 1.38 | 3.23E-04 | 7.05E-03 |
| **ENSG00000137880** | GCHFR | 2644 | 212.996171 | 1.41 | 2.16E-04 | 5.02E-03 |
| **ENSG00000173546** | CSPG4 | 1464 | 306.172904 | 1.43 | 3.56E-06 | 1.55E-04 |
| **ENSG00000105855** | ITGB8 | 3696 | 2246.94077 | 1.44 | 1.04E-27 | 4.47E-25 |
| **ENSG00000137752** | CASP1 | 834 | 168.380152 | 1.44 | 3.23E-04 | 7.05E-03 |
| **ENSG00000113924** | HGD | 3081 | 228.950273 | 1.46 | 2.77E-04 | 6.23E-03 |
| **ENSG00000166578** | IQCD | 115811 | 151.178718 | 1.50 | 4.04E-04 | 8.45E-03 |
| **ENSG00000106537** | TSPAN13 | 27075 | 1331.83328 | 1.50 | 7.90E-20 | 2.12E-17 |
| **ENSG00000116774** | OLFML3 | 56944 | 255.45674 | 1.50 | 4.42E-06 | 1.86E-04 |
| **ENSG00000120899** | PTK2B | 2185 | 168.708952 | 1.52 | 1.66E-04 | 4.03E-03 |
| **ENSG00000123689** | G0S2 | 50486 | 7380.03113 | 1.53 | 1.33E-62 | 1.81E-59 |
| **ENSG00000081059** | TCF7 | 6932 | 438.888155 | 1.54 | 2.73E-08 | 1.99E-06 |
| **ENSG00000256235** | SMIM3 | 85027 | 2121.20873 | 1.54 | 3.68E-33 | 2.15E-30 |
| **ENSG00000111885** | MAN1A1 | 4121 | 226.222439 | 1.55 | 9.29E-06 | 3.57E-04 |
| **ENSG00000173432** | SAA1 | 6288 | 43143.3076 | 1.61 | 9.32E-118 | 7.62E-114 |
| **ENSG00000149557** | FEZ1 | 9638 | 701.734389 | 1.62 | 1.47E-14 | 2.40E-12 |
| **ENSG00000205362** | MT1A | 4489 | 153.161307 | 1.62 | 1.52E-04 | 3.78E-03 |
| **ENSG00000101955** | SRPX | 8406 | 518.173214 | 1.67 | 2.78E-11 | 3.18E-09 |
| **ENSG00000067141** | NEO1 | 4756 | 1054.57348 | 1.67 | 4.30E-19 | 1.13E-16 |
| **ENSG00000137501** | SYTL2 | 54843 | 191.836934 | 1.68 | 2.18E-05 | 7.25E-04 |
| **ENSG00000066629** | EML1 | 2009 | 163.935658 | 1.71 | 4.26E-05 | 1.27E-03 |
| **ENSG00000182492** | BGN | 633 | 352.111627 | 1.72 | 2.31E-09 | 2.06E-07 |
| **ENSG00000171303** | KCNK3 | 3777 | 171.113577 | 1.73 | 1.90E-04 | 4.51E-03 |
| **ENSG00000141576** | RNF157 | 114804 | 134.300113 | 1.84 | 4.46E-05 | 1.32E-03 |
| ENSG00000279519 | |  | 92.6531848 | 1.87 | 4.69E-04 | 9.51E-03 |
| **ENSG00000134339** | SAA2 | 6289 | 5265.05987 | 1.88 | 9.09E-87 | 2.48E-83 |
| **ENSG00000148344** | PTGES | 9536 | 555.857198 | 1.92 | 2.71E-15 | 4.77E-13 |
| **ENSG00000248905** | FMN1 | 342184 | 108.81129 | 1.95 | 1.48E-04 | 3.68E-03 |
| **ENSG00000017483** | SLC38A5 | 92745 | 108.276229 | 1.97 | 6.33E-05 | 1.79E-03 |
| **ENSG00000165949** | IFI27 | 3429 | 2762.05877 | 1.98 | 2.44E-44 | 2.10E-41 |
| **ENSG00000126970** | ZC4H2 | 55906 | 213.469288 | 2.04 | 8.98E-09 | 7.17E-07 |
| **ENSG00000161544** | CYGB | 114757 | 740.01927 | 2.10 | 2.21E-23 | 7.36E-21 |
| **ENSG00000175471** | MCTP1 | 79772 | 420.343958 | 2.26 | 3.12E-13 | 4.37E-11 |
| **ENSG00000145794** | MEGF10 | 84466 | 81.6989343 | 2.29 | 4.15E-04 | 8.65E-03 |
| **ENSG00000158714** | SLAMF8 | 56833 | 98.0547315 | 2.29 | 1.15E-05 | 4.27E-04 |
| **ENSG00000162595** | DIRAS3 | 9077 | 495.266525 | 2.31 | 1.99E-16 | 3.83E-14 |
| **ENSG00000153294** | ADGRF4 | 221393 | 93.4079558 | 2.35 | 1.07E-04 | 2.78E-03 |
| **ENSG00000109906** | ZBTB16 | 7704 | 78.4343216 | 2.41 | 6.24E-05 | 1.77E-03 |
| **ENSG00000100344** | PNPLA3 | 80339 | 120.645362 | 2.53 | 1.34E-07 | 8.42E-06 |
| **ENSG00000138792** | ENPEP | 2028 | 62.6997558 | 2.57 | 9.31E-05 | 2.48E-03 |
| **ENSG00000172156** | CCL11 | 6356 | 55.7259232 | 2.61 | 2.45E-04 | 5.62E-03 |
| **ENSG00000183876** | ARSI | 340075 | 279.739311 | 2.63 | 6.41E-16 | 1.19E-13 |
| **ENSG00000063438** | AHRR | 57491 | 86.6491091 | 2.68 | 7.21E-06 | 2.83E-04 |
| **ENSG00000172061** | LRRC15 | 131578 | 67.9150662 | 2.70 | 2.71E-05 | 8.67E-04 |
| **ENSG00000198768** | APCDD1L | 164284 | 96.624931 | 2.70 | 1.68E-06 | 8.05E-05 |
| **ENSG00000157613** | CREB3L1 | 90993 | 742.720372 | 2.70 | 6.29E-38 | 3.96E-35 |
| **ENSG00000107562** | CXCL12 | 6387 | 446.078907 | 2.72 | 7.22E-20 | 1.97E-17 |
| **ENSG00000107731** | UNC5B | 219699 | 56.6056621 | 2.74 | 1.54E-04 | 3.81E-03 |
| **ENSG00000129757** | CDKN1C | 1028 | 762.844029 | 2.74 | 5.60E-41 | 4.16E-38 |
| **ENSG00000108342** | CSF3 | 1440 | 1192.13535 | 2.83 | 6.48E-63 | 9.63E-60 |
| ENSG00000274248 | |  | 65.4732541 | 2.91 | 2.81E-04 | 6.29E-03 |
| **ENSG00000137440** | FGFBP1 | 9982 | 222.724767 | 2.92 | 2.75E-10 | 2.83E-08 |
| **ENSG00000139329** | LUM | 4060 | 238.685936 | 2.98 | 1.73E-14 | 2.74E-12 |
| **ENSG00000124191** | TOX2 | 84969 | 850.770331 | 3.02 | 1.70E-44 | 1.55E-41 |
| **ENSG00000168772** | CXXC4 | 80319 | 37.7094051 | 3.11 | 3.62E-04 | 7.75E-03 |
| **ENSG00000169122** | FAM110B | 90362 | 48.8231407 | 3.27 | 7.19E-05 | 1.98E-03 |
| **ENSG00000184012** | TMPRSS2 | 7113 | 375.879216 | 3.32 | 2.78E-28 | 1.26E-25 |
| **ENSG00000111058** | ACSS3 | 79611 | 137.024147 | 3.54 | 4.66E-09 | 3.97E-07 |
| **ENSG00000233608** | TWIST2 | 117581 | 377.377504 | 3.55 | 2.36E-32 | 1.21E-29 |
| **ENSG00000090539** | CHRD | 8646 | 138.635036 | 3.68 | 1.16E-13 | 1.68E-11 |
| **ENSG00000197928** | ZNF677 | 342926 | 62.7502606 | 3.70 | 1.95E-05 | 6.61E-04 |
| **ENSG00000170801** | HTRA3 | 94031 | 28.722987 | 3.77 | 3.42E-04 | 7.38E-03 |
| ENSG00000232679 | |  | 51.1261443 | 3.80 | 1.34E-06 | 6.60E-05 |
| **ENSG00000269416** | LINC01224 | 104472717 | 42.5611048 | 3.91 | 1.88E-05 | 6.42E-04 |
| **ENSG00000100055** | CYTH4 | 27128 | 35.663888 | 4.09 | 4.30E-05 | 1.28E-03 |
| ENSG00000229116 | |  | 29.0788266 | 4.26 | 3.11E-04 | 6.84E-03 |
| **ENSG00000165474** | GJB2 | 2706 | 225.952293 | 4.28 | 5.43E-27 | 2.22E-24 |
| **ENSG00000174792** | ODAPH | 152816 | 250.692768 | 5.16 | 6.30E-30 | 3.03E-27 |
| ENSG00000227706 | |  | 77.7479364 | 5.47 | 6.16E-12 | 7.41E-10 |
| **ENSG00000121743** | GJA3 | 2700 | 13.4088016 | 7.22 | 3.46E-04 | 7.44E-03 |
| **ENSG00000124785** | NRN1 | 51299 | 40.5003546 | 7.36 | 7.00E-06 | 2.75E-04 |
| ENSG00000273001 | |  | 20.6171374 | 7.84 | 2.33E-05 | 7.62E-04 |
| **ENSG00000022556** | NLRP2 | 55655 | 235.647975 | 7.90 | 3.51E-23 | 1.15E-20 |
| **ENSG00000213988** | ZNF90 | 7643 | 24.5557377 | 8.09 | 9.05E-06 | 3.49E-04 |
| **ENSG00000158321** | AUTS2 | 26053 | 26.9489694 | 8.23 | 2.59E-06 | 1.16E-04 |
